# Recent Loss of Self-Incompatibility by Degradation of the Male Component in Allotetraploid *Arabidopsis kamchatica*

**DOI:** 10.1371/journal.pgen.1002838

**Published:** 2012-07-26

**Authors:** Takashi Tsuchimatsu, Pascal Kaiser, Chow-Lih Yew, Julien B. Bachelier, Kentaro K. Shimizu

**Affiliations:** Institute of Evolutionary Biology and Environmental Studies, Institute of Plant Biology, and Zurich-Basel Plant Science Center, University of Zurich, Zurich, Switzerland; University of Georgia, United States of America

## Abstract

The evolutionary transition from outcrossing to self-fertilization (selfing) through the loss of self-incompatibility (SI) is one of the most prevalent events in flowering plants, and its genetic basis has been a major focus in evolutionary biology. In the Brassicaceae, the SI system consists of male and female specificity genes at the *S*-locus and of genes involved in the female downstream signaling pathway. During recent decades, much attention has been paid in particular to clarifying the genes responsible for the loss of SI. Here, we investigated the pattern of polymorphism and functionality of the female specificity gene, the *S*-locus receptor kinase (*SRK*), in allotetraploid *Arabidopsis kamchatica*. While its parental species, *A. lyrata* and *A. halleri*, are reported to be diploid and mainly self-incompatible, *A. kamchatica* is self-compatible. We identified five highly diverged *SRK* haplogroups, found their disomic inheritance and, for the first time in a wild allotetraploid species, surveyed the geographic distribution of *SRK* at the two homeologous *S*-loci across the species range. We found intact full-length *SRK* sequences in many accessions. Through interspecific crosses with the self-incompatible and diploid congener *A. halleri*, we found that the female components of the SI system, including *SRK* and the female downstream signaling pathway, are still functional in these accessions. Given the tight linkage and very rare recombination of the male and female components on the *S*-locus, this result suggests that the degradation of male components was responsible for the loss of SI in *A. kamchatica*. Recent extensive studies in multiple Brassicaceae species demonstrate that the loss of SI is often derived from mutations in the male component in wild populations, in contrast to cultivated populations. This is consistent with theoretical predictions that mutations disabling male specificity are expected to be more strongly selected than mutations disabling female specificity, or the female downstream signaling pathway.

## Author Summary

Since Charles Darwin recognized the extraordinary diversity of plant mating systems, deciphering their origins has been a central theme for evolutionary biologists. Among various sexual systems, the evolution of self-fertilization (selfing) from cross-fertilization is one of the most frequent transitions in flowering plants, but the genetic basis responsible for these changes is still poorly understood. Here, we have focused on the evolution of selfing in the Brassicaceae, where cross-fertilization is usually enforced by a self-incompatibility (SI) system, determining specific recognition between the pistil (female component) and the pollen (male component). Through sequencing analysis and crossing experiments, we studied the genetic changes leading to the loss of SI in the selfing species *Arabidopsis kamchatica*. We found that the female components of the SI system remain functional in many accessions, suggesting that degradation of the male component was responsible for the loss of SI. Recent studies in the Brassicaceae suggest that such a male-driven loss of SI is more common in wild than in cultivated populations. Furthermore, this pattern is consistent with theories predicting that mutations disabling male specificity have a higher probability of leading to successful mating and are thus more likely to spread than those disabling female specificity.

## Introduction

The evolutionary transition from outcrossing to predominant self-fertilization (selfing) is one of the most prevalent events in flowering plants [1]–[4]. Although increased homozygosity caused by selfing often leads to reduced fitness in the offspring (inbreeding depression), the inherent transmission advantage would favor selfing [5], [6]. This is because selfers can transmit gametes in three ways—as both the ovule and pollen donor for their own selfed progeny and as the pollen donor for outcrossed progeny—whereas outcrossers cannot serve as pollen donors for their selfed progeny. Thus, an allele promoting selfing has a 3∶2 transmission advantage relative to an allele promoting outcrossing. Selfing would also be advantageous, because selfers can reproduce when pollinators or mates are scarce (“reproductive assurance” [6]–[9]). Theory suggests that selfing should evolve when these advantages outweigh the costs of inbreeding depression [6], [10], [11].

In many flowering plants, predominant selfing evolved through the loss of self-incompatibility (SI) [2], [3]. SI systems have evolved multiple times in diverse lineages of flowering plants. They generally consist of female and male specificity genes at the *S*-locus and other genes involved in signaling pathways [12]–[14]. In SI species, the *S*-locus region is subject to negative frequency-dependent selection, which is a classic example of multiallelic balancing selection [15]. The molecular basis of the SI system has been studied extensively and is well characterized in the Brassicaceae. Here, female specificity is known to be determined by the *S*-locus receptor kinase (SRK) and male specificity is determined by the *S-*locus cysteine-rich protein (SCR; also known as *S*-locus protein 11, SP11). SRK is a transmembrane serine/threonine receptor kinase that functions on the stigma, and SCR is a small cysteine-rich protein present in the pollen coat that acts as a ligand for the SRK receptor protein [12]–[14], [16]–[19]. *SRK* and *SCR* are tightly linked at the *S-*locus, where dozens of highly divergent sequence groups, called *S*-haplogroups (or *S*-haplotypes or *S*-alleles), are segregating. *S*-haplogroups confer specificity in self-recognition: direct interaction between SRK and SCR of the same *S*-haplogroup leads to the inhibition of pollen germination at the stigma [13], [20]. In the Brassicaceae, several genes involved in the female downstream signaling pathway of SRK have also been reported, such as *M-locus protein kinase* (*MLPK*) and *ARM-repeat containing 1* (*ARC1*) [13], [21]–[23].

The genetic basis for the recurrent loss of SI has been a major focus from both theoretical and empirical viewpoints [4], [24]–[29]. In particular, much attention has been paid to clarifying which mutations are responsible for the loss of SI, and whether these mutations are in the female specificity gene, the male specificity gene or in downstream signaling pathways [4], [24]–[29]. With the advantage of a suite of molecular tools, the most extensively studied species in the Brassicaceae is the diploid self-compatible *Arabidopsis thaliana* [4], [29]–[42]. Whereas a number of mutations disabling the male and female components have been identified in wild accessions of *A. thaliana* [30], [33], [36], [38], [39], many accessions still retain full-length and expressed *SRK* sequences [38], [41]. Importantly, interspecific crossings using the self-incompatible *Arabidopsis halleri* revealed that some *A. thaliana* accessions, including Wei-1, retain a functioning female SI reaction, demonstrating that all female components including *SRK* and the female downstream signaling pathway are still functional [41]. In addition, Tsuchimatsu et al. [41] reported that a 213-base-pair (bp) inversion (or its derivative haplotypes) in the *SCR* gene is found in 95% of European accessions. When the 213-bp inversion in *SCR* was inverted and expressed in transgenic Wei-1 plants, the functional SCR restored the SI reaction. These results suggested that degradation of *SCR* (the male specificity gene) was primarily responsible for the evolutionary loss of SI of the *S*-haplogroup in European *A. thaliana*, while other mutations at genes involved in the downstream signaling pathway might have contributed to some extent [35], [39].

To understand whether such mutations in the male components of the *S-*locus are common in the recurrent evolution of self-compatibility in the Brassicaceae, empirical examples need to be investigated in other self-compatible species. In addition to *A. thaliana*, there are a few reports on the pattern of polymorphism at the *S-*locus in self-compatible species in Brassicaceae, such as *Capsella rubella* [43] and *Leavenworthia alabamica* [44]. However, the genetic and molecular bases responsible for the loss of SI are still unknown in these species. A major obstacle to charting the history of the *S-*locus in self-compatible species has been in distinguishing the primary inactivating mutation from subsequent decay of the nonfunctional *S*-haplogroups by further mutations. This is because all genes involved in this signaling pathway are expected to be released from selection pressure and to evolve neutrally once the SI system ceases to function [4], [28], [39], [41], although pleiotropy of these genes could play a role in maintaining the functionality of the signaling pathway [42], [45]. To study the primary mutations, a powerful approach would be to combine experiments confirming gene function with population genetic analyses finding gene-disruptive mutations.

*Arabidopsis kamchatica* would be a good model system to address this issue. It is a self-compatible species, and originated through allopolyploidization of two species, *A. halleri* and *A. lyrata*, which are reported to be diploid [46]–[51]. Shimizu-Inatsugi et al. [48] reported that multiple haplotypes of nuclear and chloroplast sequences of *A. kamchatica* are identical to those of their parental species, indicating that multiple diploid individuals of *A. halleri* and *A. lyrata* contributed to the origin of *A. kamchatica*. In particular, *A. kamchatica* and *A. halleri* share four chloroplast haplotypes, strongly suggesting that at least four diploid individuals of *A. halleri* contributed independently to the multiple origins of *A. kamchatica* [48]. The two parental species are predominantly self-incompatible, although some of North American populations of *A. lyrata* are known to be self-compatible [26], [29]. Their SI systems have been extensively studied [26], [52]–[61]. Most of these studies have focused on *SRK* to characterize *S*-haplogroups, because novel *SRK* haplogroups can be isolated relatively easily by using PCR primers that were designed in the conserved regions of *SRK* [52], [55], while much fewer *SCR* sequences have been isolated because of its extreme polymorphism [30], [40], [62], [63]. To date, 40 and 30 *SRK* haplogroups have been reported in *A. lyrata* and *A. halleri*, respectively, and studies of nucleotide polymorphisms and divergence using large sets of *SRK* sequences revealed various characteristics of the *S-*locus, such as the spatial distribution of *S-*haplogroups, complex dominance interactions and transspecific sharing of *S-*haplogroups among species [55]–[57], [59], [60], [64]. The wealth of knowledge available on these parental species enabled us to investigate nucleotide polymorphisms of the *S-*locus in self-compatible *A. kamchatica*. In addition, *A. kamchatica* would be a novel model to investigate the mechanism underlying the loss of SI in polyploid species. The relationship between self-compatibility and polyploidy has been debated for more than 60 years, as it is argued that polyploids have higher selfing rates than their diploid relatives [1], [65] (but see also [66] for controversy). Hypotheses have been proposed to explain this association, such as: (1) self-compatible individuals in polyploids would not suffer from limitation of mates of the same ploidy level [1], [66]–[70]; and (2) inbreeding depression would be reduced by having multiple gene copies [10], [66], [71], [72]. Despite numerous ecological and evolutionary studies, the molecular mechanisms underlying the evolution of self-compatibility of polyploid species are still poorly understood.

To understand the mechanisms underlying the loss of SI in *A. kamchatica*, we first isolated *SRK* haplogroups from *A. kamchatica* by examining 48 populations across its distribution range (Table S1). Based on the analyses of nucleotide divergence from parental species and the distribution of *SRK* haplogroups with respect to population structure, we studied how *S*-haplogroups in *A. kamchatica* have originated from the parental species. To understand the approximate timescale of this evolutionary event, we also estimated the divergence time of *A. kamchatica* from its parental species based on the nucleotide divergence of multiple nuclear genes other than *SRK.* This is because speciation time would be used as the upper boundary of the time estimate of the evolution of self-compatibility in a species, when the progenitor species was self-incompatible [43], [73]. We tested the function of *SRK* haplogroups through interspecific crossing with self-incompatible *A. halleri*, and confirmed the disomic inheritance and allelic relationships of *SRK* haplogroups by segregation analyses in experimental and natural populations of *A. kamchatica*. Most importantly, our interspecific crossing with *A. halleri* also indicated the retained function of the female component of SI in *A. kamchatica*, suggesting that the degradation of male components was responsible for the loss of SI. We suggest that the degradation of male components among the Brassicaceae might represent a general trend in the evolution of self-compatibility in wild populations in contrast to cultivated populations.

## Results

### Five highly diverged *SRK*-like sequences in *A. kamchatica* revealed by PCR–based screening

**Figure 1:**
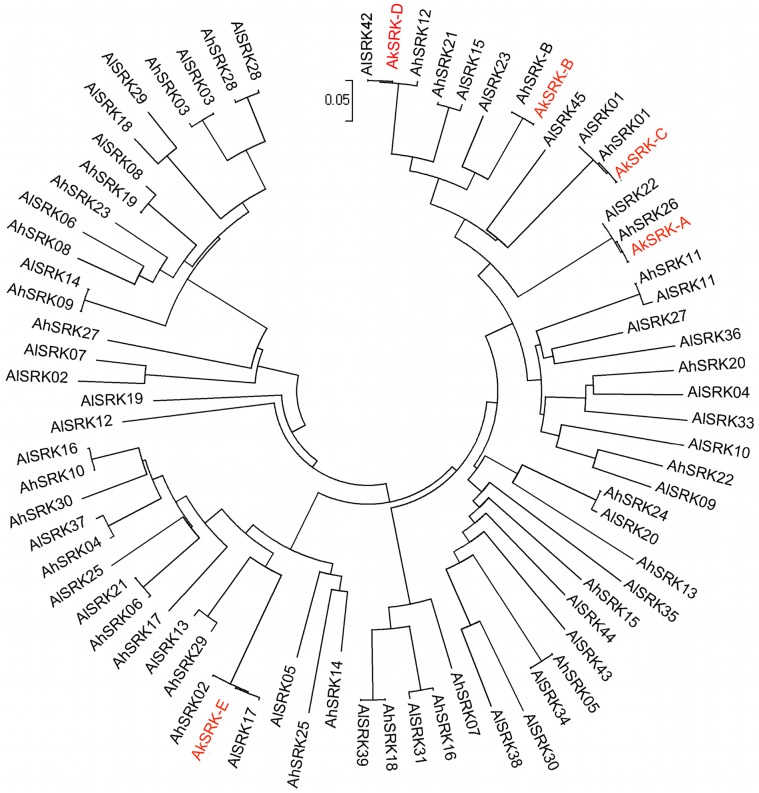
Phylogeny of 76 *SRK* sequences of *A. halleri*, *A. lyrata*, and *A. kamchatica*. This phylogeny was obtained by the neighbor-joining method on pairwise proportions of nucleotide divergence. In total, 266 nucleotide positions were used. The evolutionary distances were computed using the Kimura two-parameter method. The tree includes 31 *SRK* sequences of *A. halleri* (*AhSRK*), 40 *SRK* sequences of *A. lyrata* (*AlSRK*) and five *SRK* sequences of *A. kamchatica* (*AkSRK*). See Table S10 for the accession numbers of these sequences deposited in GenBank. *SRK* sequences of *A. halleri* and *A. lyrata* are shown in black, and those of *A. kamchatica* are shown in red.

Through PCR-based screening, we obtained five partial *SRK*-like sequences from *A. kamchatica*, named *AkSRK-A*, *AkSRK-B*, *AkSRK-C*, *AkSRK-D* and *AkSRK-E*. Our five *SRK* sequences were aligned with *S*-domain sequences available for *SRK* from *A. halleri* and *A. lyrata* [52], [54], [55], [58], [59], [61], and a phylogenetic tree including a total of 76 *SRK* sequences was generated using the neighbor-joining method (Figure 1). While previous studies reported that the *SRK* haplogroups are transspecifically shared among *A. halleri*, *A. lyrata* and *A. thaliana* [59], [62], the tree clearly shows that the *SRK* haplogroups are also transspecifically shared between *A. kamchatica*, *A. halleri* and *A. lyrata* (Figure 1).

In *A. lyrata* and *A. halleri*, *SRK* sequences that presumably share the same specificities are highly homologous, while sequences with different specificities show at most 91–92% nucleotide sequence identity [59], [74]. Here, four out of the five *A. kamchatica SRK* sequences (*AkSRK-A*, *AkSRK-C*, *AkSRK-D* and *AkSRK-E*) showed more than 98% sequence identity to *SRK* sequences previously reported in both *A. halleri* and *A. lyrata* (Figure 1), suggesting that they also share specificities with the corresponding *A. halleri* and *A. lyrata SRK* sequences. In contrast, no previously reported sequence showed any particularly high similarity with *AkSRK-B*. The most similar ones were *AhSRK12* (81% identity over 576 bp) and *AlSRK23* (87% identity over 558 bp), suggesting that they are unlikely to share specificity with *AkSRK-B*. Using specific primers for *AkSRK-B*, we successfully amplified a sequence from an *A. halleri* plant (lowland habitat in Western Honshu, Japan; No. 61 in Table S1) that showed 100% sequence identity to *AkSRK-B* of *A. kamchatica* and, as shown later by the interspecific crosses, they shared the functional specificity of SI. This newly discovered *A. halleri* ortholog was named *AhSRK-B* (Figure 1). Using specific primers, we also isolated orthologous sequences of *AkSRK-A* and *AkSRK-C* from *A. halleri*, which were nearly identical to *AhSRK26* and *AhSRK01*, respectively (Figure S1). These sequences were named *AhSRK26-*Ibuki and *AhSRK01-*Ibuki, respectively (see the section “Retained full-length *SRK* sequences as well as multiple gene-disruptive mutations” for details).

### Nucleotide divergence of *AkSRK* from orthologous genes of the parental species *A. halleri* and *A. lyrata*

**Figure 2:**
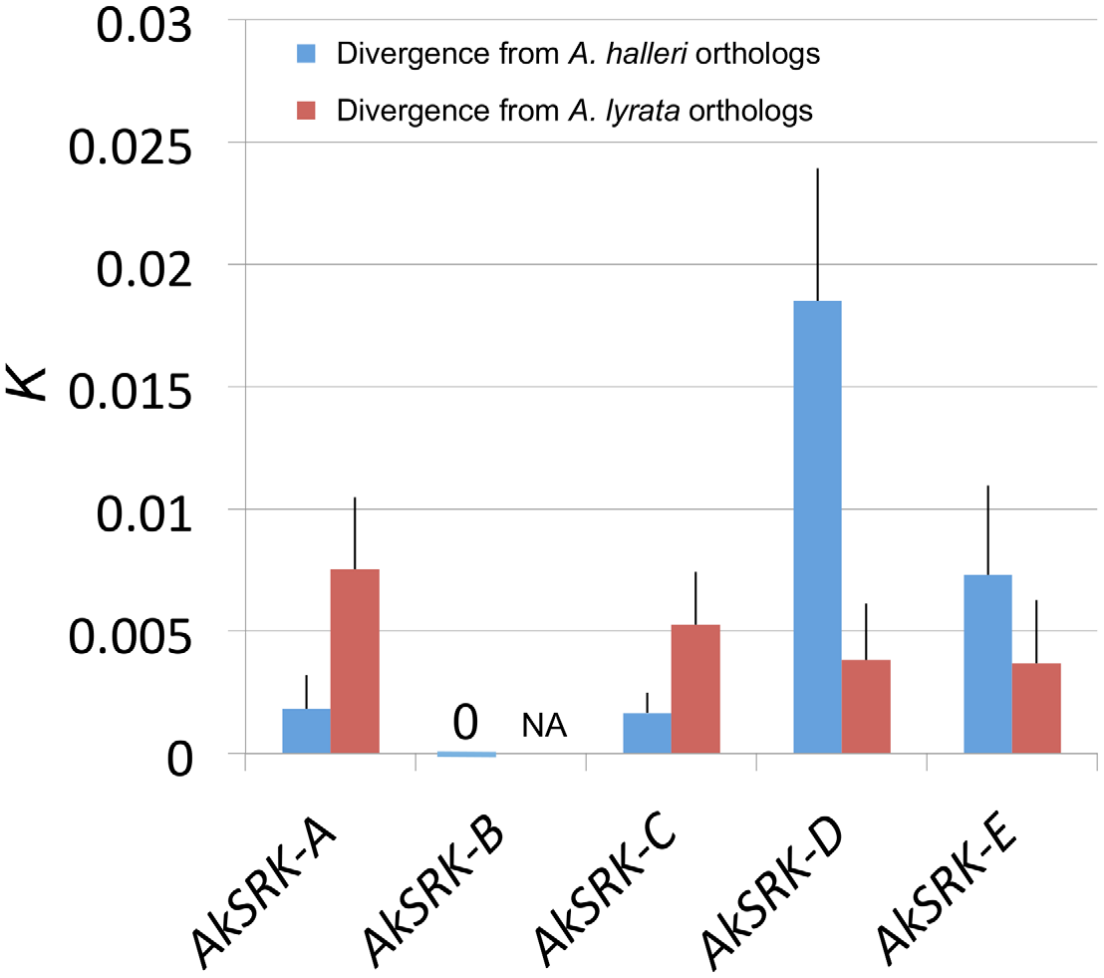
Nucleotide divergence of *AkSRK* of five *S*-haplogroups in *A. kamchatica* from two parental species. All estimates were Jukes–Cantor corrected. Bars indicate standard errors. Sequence lengths compared were: 935 bp (*AkSRK-A*), 3773 bp (*AkSRK-B*), 1067 bp (*AkSRK-C*), 555 bp (*AkSRK-D*) and 550 bp (*AkSRK-E*). *AkSRK-B* was 100% identical to *AhSRK-B* throughout the coding sequence (see also Figure 5). Because *AkSRK-B* belongs to a newly identified *S*-haplogroup (see Results), orthologous sequences were not yet reported from *A. lyrata*, while *AhSRK-B* from *A. halleri* was found in this study. Thus, nucleotide divergence from *A. halleri* only is shown for *AkSRK-B*. See also Table S2 for details.

Despite their transspecificity, several species-specific nucleotide substitutions have been reported within the same *S*-haplogroups in *A. lyrata* and *A. halleri*, respectively [61]. Thus, to obtain insight into which parental species the *SRK*-like sequences found in *A. kamchatica* were derived from, we compared the nucleotide divergence of *SRK*-like sequences of *A. kamchatica* with corresponding orthologous genes from *A. halleri* and *A. lyrata*. *AkSRK-A* and *AkSRK-C* were closer to their orthologs of *A. halleri* (*AhSRK26* and *AhSRK01*, respectively) than to those of *A. lyrata* (*AlSRK22 and AlSRK01*, respectively) (Figure 2; Figure S1; Table S2). Conversely, *AkSRK-D* and *AkSRK-E* showed higher sequence identity to orthologs from *A. lyrata* (*AlSRK42* and *AlSRK17*, respectively) (Figure 2; Figure S1; Table S2). These results suggest that *AkSRK-A* and *AkSRK-C* are derived from *A. halleri* and that *AkSRK-D* and *AkSRK-E* are derived from *A. lyrata*. In addition, because we found that *AhSRK-B* in *A. halleri* showed 100% identity to *AkSRK-B* in *A. kamchatica*, *AkSRK-B* is most likely derived from *A. halleri*. Based on this pattern of nucleotide divergence from the parental species, mutually exclusive distribution of *SRK* haplogroups and a segregation analysis in the F_2_ population (see below), hereafter we denote *AkSRK-A*, *AkSRK-B* and *AkSRK-C* as the “*A. halleri-*derived *SRK*” and *AkSRK-D* and *AkSRK-E* as the “*A. lyrata-*derived *SRK*” (see below and Discussion).

### Distribution and frequency of five *S*-haplogroups revealed by PCR–based genotyping

**Figure 3:**
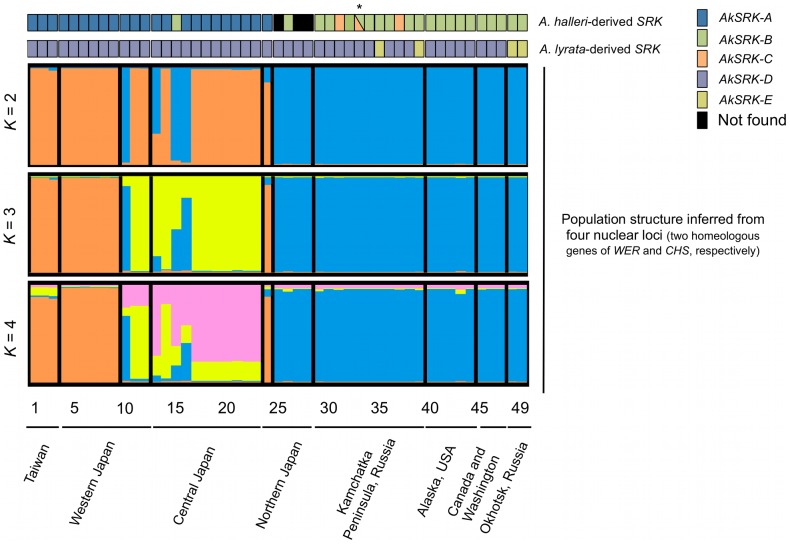
The geographic distribution of *S*-haplogroups and population structure in *A. kamchatica*. Results of PCR-based genotyping of *SRK*, with inference of population structure based on four loci (two homeologous genes each of nuclear *WER* and *CHS* genes) for the clustering of *K* = 2, 3 and 4, by the Bayesian clustering algorithm implemented in InStruct software (see Figure S2 and Table S1). Numbers below the diagrams correspond to the accessions listed in Table S1. Triangles in accession No. 33 (marked with an asterisk) indicate that *AkSRK-B* and *AkSRK-C*, as well as *AkSRK-D*, were found in a single individual. We confirmed these results using the software STRUCTURE instead of InStruct (see Text S1 and Figure S5 for details).

**Figure 4:**
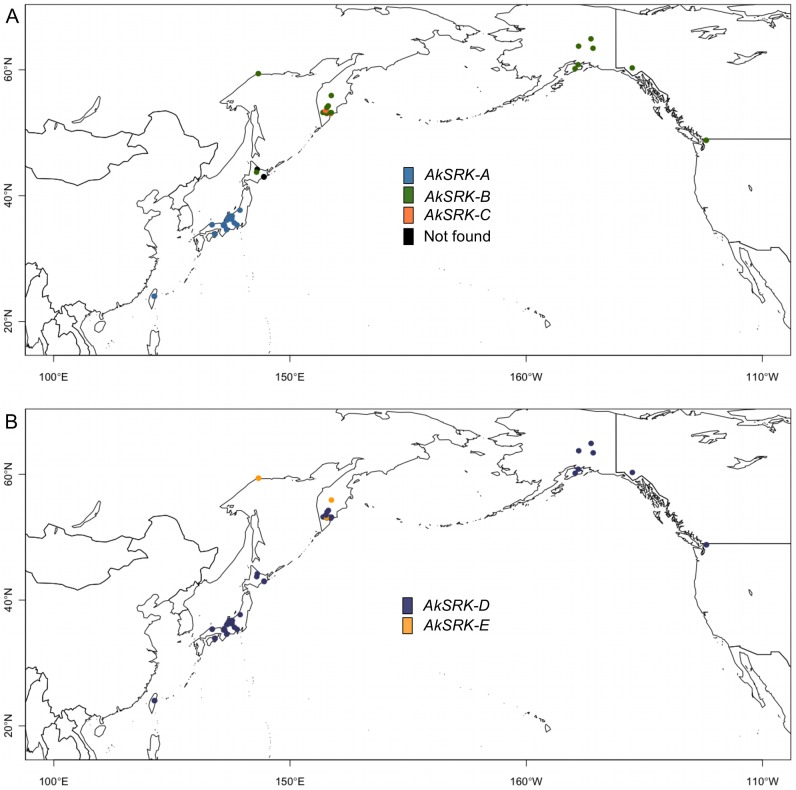
Geographic distribution of *S*-haplogroups in *A. kamchatica*. (A) Geographic distribution of *AkSRK-A* (blue), *AkSRK-B* (green) and *AkSRK-C* (orange). Black circles: not found. (B) Geographic distribution of *AkSRK-D* (purple) and *AkSRK-E* (dark yellow).

Using PCR-based genotyping of *SRK* haplogroups, we investigated the frequencies and geographic distribution of the five *S-*haplogroups identified in this study. Altogether, 49 accessions from 46 populations were genotyped by primer pairs that could specifically amplify *AkSRK-A*, *AkSRK-B*, *AkSRK-C*, *AkSRK-D* or *AkSRK-E* (Figure 3; Figure 4; Table S1; see Table S3 for the primer pairs used). Two copies of *SRK* were amplified from all accessions except those from Hokkaido in Japan that had only one copy (Nos. 25, 27 and 28), and one from Kamchatka in Russia that had three copies (No. 33). This finding is consistent with reports that *A. kamchatica* is a self-compatible allotetraploid, which usually harbors two homeologs from the parental species, supposing rare heterozygosity because of selfing and rare duplication [46], [48].

The distributions of *AkSRK-A*, *AkSRK-B* and *AkSRK-C* were mutually exclusive except for one accession (No. 33, see below) and showed a strong geographic structure. *AkSRK-A* and *AkSRK-B* were found in about half of our samples (46.9% and 41.8%, respectively; Figure 3; Figure 4); all *AkSRK-A* were found in the southwestern part of the distribution range of the species (Taiwan and Japan), whereas *AkSRK-B* was mainly located in the northern and eastern part of the species range (North America and the Kamchatka Peninsula in Russia; Figure 3; Figure 4; Table S1). In contrast, the frequency of *AkSRK-C* was lower than those of *AkSRK-A* and *AkSRK-B* (5.1%; Figure 3; Figure 4), and was found only in three accessions from the Kamchatka Peninsula (Figure 3; Figure 4). One of them (No. 33) harbored both *AkSRK-B* and *AkSRK-C*, indicating heterozygosity or duplication of the *A. halleri*-derived *SRK*, *AkSRK-B* and *AkSRK-C* sequences. We further genotyped nine additional individuals in that population (Table S4) and found that *AkSRK-B* and *AkSRK-C* segregated at intermediate frequencies (*AkSRK-B*: 0.55; *AkSRK-C*: 0.45). This is consistent with the hypothesis that No. 33 is a rare heterozygote of *AkSRK-B* and *AkSRK-C*, because it would occasionally arise even by rare outcrossing events under predominant selfing, in particular when the allele frequencies were intermediate. Moreover, the distributions of *AkSRK-D* and *AkSRK-E* were completely mutually exclusive in our 49 samples and *AkSRK-D* was nearly fixed (91.8% frequency; Figure 3; Figure 4).

The geographic distribution of the *A. halleri*-derived *S*-haplogroups is concordant with the population structure inferred from polymorphisms of four other nuclear loci (two homeologous genes each of *WER* and *CHS*) (Figure 3). The high values of the mean posterior probability of data ln *P*(*X*|*K*), *ΔK* and the symmetric similarity coefficient supported the clustering of *K* = 2, which reflect the spatial structure of the distribution well (Figure 3; Figure S2; Table S1). The distributions of *A. halleri*-derived *S*-haplogroups—*AkSRK-A*, *AkSRK-B* and *AkSRK-C*—are significantly correlated with the population structure (Cramer's coefficient: 1; *p* = 7.62×10^−12^); that is, most of accessions bearing *AkSRK-A* belong to cluster 1 (orange in Figure 3) and most of others belong to cluster 2 (blue in Figure 3). This significant correlation indicates that the pattern of distribution of these *A. halleri*-derived *S*-haplogroups is consistent with the genome-wide pattern of polymorphism. The correlations were also significant with the clustering of *K* = 3 (Cramer's coefficient: 0.675; *p* = 6.37×10^−11^) and of *K* = 4 (Cramer's coefficient: 0.577; *p* = 5.39×10^−12^). In contrast, the distributions of *A. lyrata*-derived *S*-haplogroups—*AkSRK-D* and *AkSRK-E*—are not correlated significantly with the population structure (Cramer's coefficient: 0.302; *p* = 0.109). The correlations were also not significant with the clustering of *K* = 3 (Cramer's coefficient 0.194; *p* = 0.393) and of *K* = 4 (Cramer's coefficient 0.163; *p* = 0.517). In fact, the frequency of *AkSRK-D* is markedly higher than that of *AkSRK-E* and it is widespread throughout the geographically wide range, resulting in no significant correlation between these *A. lyrata*-derived *S-*haplogroups and population structure.

In *A. kamchatica* subsp. *kawasakiana* (formerly, *A. lyrata* subsp. *kawasakiana*), a previous survey of *SRK* identified two sequences, *Aly13-1* and *Aly13-22* [57]. The result of our PCR-based genotyping is consistent with those findings, as we also found *AkSRK-A* (orthologous to *Aly13-22*) in all accessions of *A. kamchatica* subsp. *kawasakiana* (Table S1; Figure 3). However, *AkSRK-C* (orthologous to *Aly13-1*) was not found in our samples of subsp. *kawasakiana* but only in subsp. *kamchatica*.

### Estimation of the divergence time from parental species

Using the nucleotide divergence estimate *K* and the synonymous substitution rate *K*s of four nuclear loci (two homeologous genes each of *CHS* and *WER*; Table S5), we estimated the divergence time of *A. kamchatica* from the parental species (Table S6). When we employed the mutation rate given by Koch et al. [75], which is based on the synonymous substitution rate calibrated by fossil records, the mean divergence time was 20,417 years (with a 95% confidence interval of 0–75,460 years). When we employed the mutation rate given by Ossowski et al. [76], estimated using mutation accumulation lines, the mean divergence time was 245,070 years (with a 95% confidence interval of 37,385–532,953 years). Overall, estimates based on the mutation rate given by Koch et al. [75] were smaller than those based on Ossowski et al. [76], although the 95% confidence intervals overlapped.

Two clusters were suggested in the analysis of population structure (Figure 3). Whereas here we estimated the divergence averaged over population clusters, it is possible that it varies between clusters, given that population structure is profoundly affected by the multiple origins of *A. kamchatica* [48]. We also calculated the clusterwise nucleotide divergence from *A. halleri* and *A. lyrata* but no significant difference from each other was found in the current dataset and clustering (Table S5).

### *AkSRK-A* and *AkSRK-B* are allelic with each other and disomically inherited

Based on the pattern of nucleotide divergence from the parental species and the mutually exclusive distribution of *SRK*, we predicted the following allelic relationships: the *A. halleri*-derived *SRK* (*AkSRK-A*, *AkSRK-B* and *AkSRK-C*) should be allelic and the *A. lyrata*-derived *SRK* (*AkSRK-D* and *AkSRK-E*) should be allelic with each other, respectively. This prediction also assumes the disomic inheritance of the *A. halleri*- and *A. lyrata*-derived *SRK*, respectively. To confirm our predictions, we investigated the pattern of segregation of the *A. halleri*-derived *SRK* (*AkSRK-A* and *AkSRK-B*) in an F_2_ population that was generated by crossing individuals bearing *AkSRK-A* and *AkSRK-D*, and individuals bearing *AkSRK-B* and *AkSRK-D* (Table 1). We genotyped 95 F_2_ individuals with specific primers for *AkSRK-A* and *AkSRK-B*, and compared the goodness-of-fit of four inheritance models: (1) disomic and allelic, (2) disomic and nonallelic, (3) tetrasomic and allelic and (4) tetrasomic and nonallelic (Table 1). We found that the observed pattern of segregation better fitted model (1), i.e., the disomic and allelic model, rather than the other three models (*p* = 0.27; Table 1). We also confirmed the amplification of *AkSRK-D* in all 95 F_2_ plants and in the F_1_ generation, which indicates that *AkSRK-D* segregates neither with *AkSRK-A* nor with *AkSRK-B* in the F_2_ population.

**Table 1:** The pattern of segregation of *AkSRK-A* and *AkSRK-B* in 95 F_2_ individuals.

	A/B	A/−	−/B	−/−	*χ* ^2^	*P*-value
Observed	53	25	17	0		
Expected under the disomic and allelic model	47.5 (2/4)	23.5 (1/4)	23.5 (1/4)	0 (0/4)	2.62	0.27
Expected under the disomic and nonallelic model	53.4 (9/16)	17.8 (3/16)	17.8 (3/16)	5.9 (1/16)	8.88	0.031
Expected under the tetrasomic and allelic model	89.7 (34/36)	2.64 (1/36)	2.64 (1/36)	0 (0/36)	92.73	<2.2×10^−16^
Expected under the tetrasomic and nonallelic model	89.8 (1225/1296)	2.57 (35/1296)	2.57 (35/1296)	0.073 (1/1296)	102.84	<2.2×10^−16^

A/B, Amplified by both the *AkSRK-A*- and the *AkSRK-B*-specific primers. A/−, Amplified only by the *AkSRK-A* specific primer. −/B, Amplified only by the *AkSRK-B-*specific primer. −/−, Amplified by neither the *AkSRK-A*- nor the *AkSRK-B*-specific primer. Expected frequencies in each category are shown in parentheses. We confirmed the amplification of *AkSRK-D* in all 95 F_2_ plants and in the F_1_ plants.

### Retained full-length *SRK* sequences as well as multiple gene-disruptive mutations

**Figure 5:**
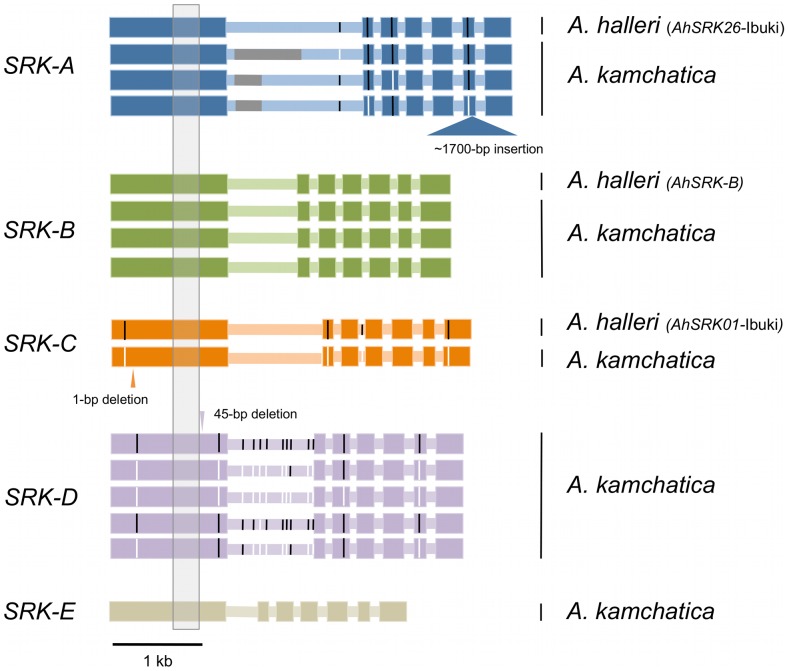
Entire coding sequences of *SRK* of five *S*-haplogroups from *A. kamchatica* and three *S*-haplogroups from *A. halleri*. Thick and thin horizontal bars indicate exons and introns, respectively. Black and white vertical bars indicate single nucleotide polymorphisms found in each haplogroup. Nucleotide sequences were not available for part of the first intron of *SRK-A* because of repetitive sequences (indicated by gray bars). The gray shaded region in exon 1 was used for generating the phylogenetic tree in Figure 1.

We isolated entire coding sequences of *SRK* from several *A. kamchatica* individuals for each haplogroup (three *AkSRK-A*, three *AkSRK-B*, one *AkSRK-C*, five *AkSRK-D* and one *AkSRK-E*; Figure 5 and Table S1). In addition, we also isolated entire coding sequences of orthologs of *AkSRK-A*, *AkSRK-B* and *AkSRK-C* from *A. halleri*. Alignment of the coding regions of *SRK* from *A. kamchatica* and *A. halleri* indicates that at least one accession of four haplogroups—A, B, D and E—retains the full-length *SRK*, without any apparent disruptive mutations such as frameshifts or inverted repeats. Furthermore, four single nucleotide polymorphisms were identified among three sequences of *AkSRK-A* and 13 among five sequences of *AkSRK-D* (Figure 5).

No obvious gene-disruptive mutations were found in the sequences of *A. halleri*, but we found three in multiple haplogroups of *A. kamchatica* (Figure 5; Figure S3). First, we found that *AkSRK-A* from an accession from Biwako, a lowland region of Japan, contained an approximately 1,700-bp insertion in exon 6. PCR-based genotyping revealed that this insertion is shared by all the accessions of *A. kamchatica* subsp. *kawasakiana* living in lowland regions in western Japan (Table S1; see Table S3 for the PCR primers used). We also found a 1-bp deletion in *AkSRK-C* from a Kamchatka accession that caused a frameshift. In addition, we found that *AkSRK-D* of an accession from mountains in central Honshu contains a 45-bp deletion in exon 1. Although this deletion does not change the reading frame, it is likely to abolish the recognition function, because it lies within the *S*-domain and encompasses one of the 12 conserved cysteine residues suggested to be important for protein structure [77], [78] (Figure 5; Figure S3; see also the section on interspecific crosses for the degraded female SI in the Murodo accession).

### Interspecific crosses between *A. halleri* and *A. kamchatica* suggest that degradation of the male components was responsible for the loss of SI

To test whether these full-length *SRK* sequences and other components involved in the female signaling pathway are functional in *A. kamchatica*, we first conducted interspecific crosses between *A. halleri* (male) and *A. kamchatica* (female). As *S*-haplogroups are shared transspecifically among *A. lyrata*, *A. halleri* and *A. kamchatica* (Figure 1), an incompatible reaction should occur even in interspecific crosses in which pollen and stigma share the same haplogroup [41]. We used the three most frequent haplogroups in *A. kamchatica*—A, B and D—as all of the accessions surveyed contain at least one of them.

**Figure 6:**
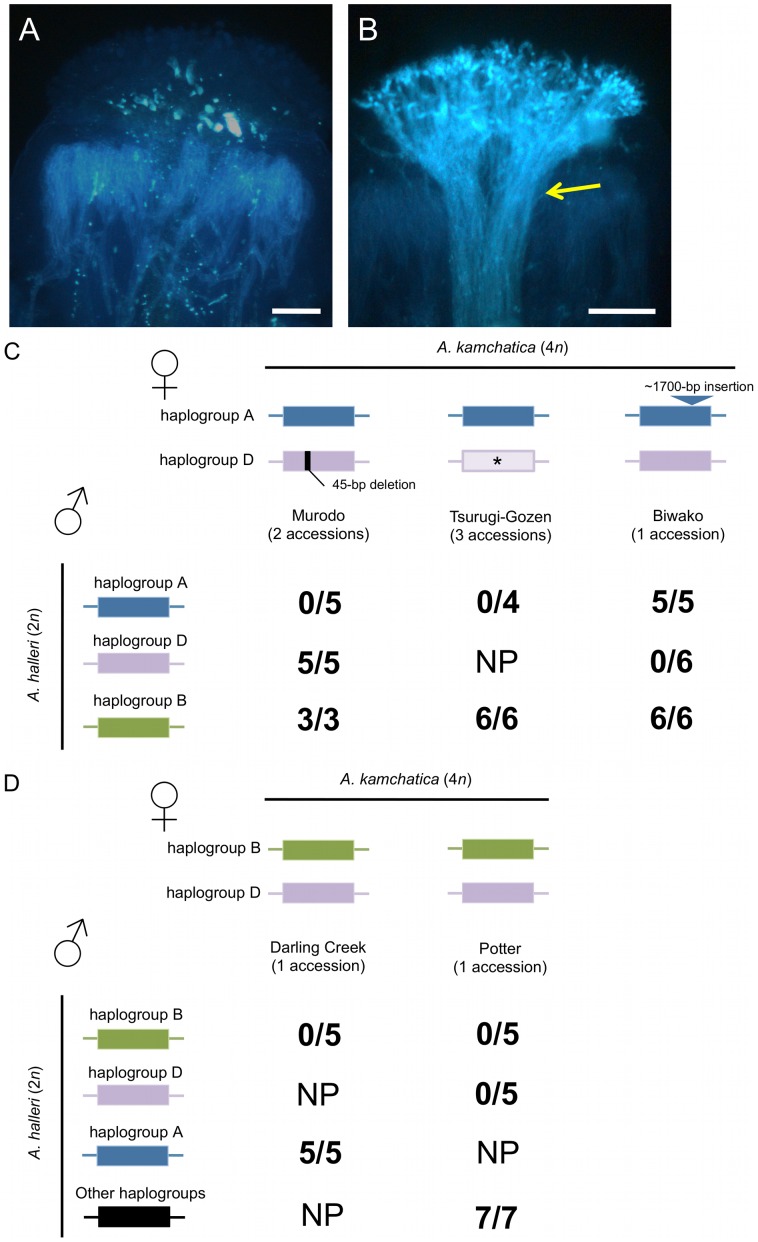
Interspecific crosses between *A. halleri* and *A. kamchatica.* (A, B) Representative incompatible (A) and compatible (B) reactions on *A. kamchatica* stigmas. Crosses were carried out between *A. halleri* bearing haplogroup B and *A. kamchatica* bearing haplogroup B (A), and between *A. halleri* not bearing haplogroup B and *A. kamchatica* bearing haplogroup B (B). A bundle of pollen tubes indicates a compatible reaction (yellow arrow). Scale bars = 0.1 mm. (C) Crosses using *A. kamchatica* from Murodo, Tsurugi-Gozen and Biwako, bearing *AkSRK-A* and *AkSRK-D*. The *AkSRK-D* sequence of Murodo has a 45-bp deletion including one of the 12 conserved cysteine residues (see text). The *AkSRK-A* sequence of Biwako has a ∼1,700-bp insertion in exon 6 (see text and Table S1). Note that full-length sequences of *AkSRK-D* from Tsurugi-Gozen were not confirmed and that crosses with *A. halleri* bearing haplogroup D were not conducted (indicated by asterisks). (D) Crosses using *A. kamchatica* from Darling Creek and Potter, bearing *AkSRK-B* and *AkSRK-D.* Two *A. halleri* plants that do not bear *SRK-A*, *SRK-B* or *SRK-D* were also used as pollen donors (indicated as “Other haplogroups”; see Methods). (C, D) Numerators denote crosses where more than 20 pollen tubes penetrated the stigma, indicating compatible reactions. Denominators show the total number of crosses conducted in each combination. NP: not performed.

In eight accessions of *A. kamchatica*, incompatible reactions were observed when pollen of *A. halleri* was used to pollinate pistils of *A. kamchatica* sharing the same haplogroups (Figure 6). These interspecific crosses verified the shared specificities of the SI system between *A. halleri* and *A. kamchatica.* More importantly, these results indicate that the female components of the SI system are functional in these accessions of *A. kamchatica*. Specifically, we found incompatible reactions in the following combinations of crosses: pollen of *A. halleri* bearing haplogroup A with pistils of *A. kamchatica* accessions bearing *AkSRK-A* from Murodo and Tsurugi-Gozen; pollen of *A. halleri* bearing haplogroup D with pistils of *A. kamchatica* bearing *AkSRK-D* from Biwako and Potter; and pollen of *A. halleri* bearing haplogroup B with pistils of *A. kamchatica* bearing *AkSRK-B* from Darling Creek and Potter. In the Potter accession, incompatible reactions were observed when haplogroups B and D of *A. halleri* were pollinated, respectively, suggesting that these haplogroups are codominant on pistils.

As control experiments, we also carried out crosses of the following combinations: (1) where the *S-*haplogroups of pollen and stigmas differ, and (2) where the *SRK* of *A. kamchatica* possessed any gene-disruptive mutations (Figure 6). In these combinations, we consistently observed compatible reactions, indicating that the reduced pollen growth observed in this experiment was caused not by interspecific reproductive isolation between *A. halleri* and *A. kamchatica*, but by the SI system.

It is worth noting that, in the Murodo accession, incompatible reactions were observed when plants were pollinated by *A. halleri* containing haplogroup A, but not by those containing haplogroup D. Because all components of the signaling pathway except for SRK are thought to be shared among different specificities, the data strongly suggest that a mutation in *AkSRK-D*—most likely the 45-bp deletion—was responsible for this decay of the female SI reaction. Likewise, compatibility of haplogroup A of the Biwako population is also attributable to a mutation in *SRK*, the approximately 1,700-bp insertion, because the functionality of the downstream signaling pathway was shown by crosses with *A. halleri* containing haplogroup D.

Because all these accessions of *A. kamchatica* are self-compatible and retain functional female components of SI including downstream signaling pathways, the data indicate that the male components of *S*-haplogroups A, B and D are not functional in these accessions. To confirm the degradation of the male components, we conducted interspecific crosses between *A. halleri* (female) and *A. kamchatica* (male), using *A. halleri* bearing haplogroups A, B or D as pistil donors and *A. kamchatica* as a pollen donor. In these combinations, we consistently observed compatible reactions, indicating that the male components of *S*-haplogroups A, B and D are not functional in these accessions of *A. kamchatica* (Figure S4). In addition, we conducted intraspecific crosses among *A. kamchatica*, with the Biwako accession as a pollen donor and the Murodo accession, which retains the functional female specificity of haplogroup A, as a pistil donor (Table S7). Incompatible reactions were not observed, thus excluding the possibility that the male components of haplogroup A of the Biwako accession remain functional. Similarly, we found that the male components of haplogroup D of Murodo are also not functional. All these data confirm that the male components of haplogroups A, B and D are not functional in these accessions of *A. kamchatica*. These nonfunctional male components and functional female components including downstream signaling pathways suggest that degradation of the male components was primarily responsible for their loss of SI. We do not exclude the possibility that recombination events between the male and female components on the *S*-locus may have also been involved, although their occurrence is reported to be very rare (reviewed in [56]).

## Discussion

### *A. halleri–* and *A. lyrata*–derived homeologs of the *S-*locus in allotetraploid *A. kamchatica*

In this study, we identified the full-length *SRK* sequences of five *S*-haplogroups in *A. kamchatica* (Figure 1). Through interspecific crosses with *A. halleri*, we confirmed that the intact *SRK* sequences of the three most frequent *S*-haplogroups in our dataset—*AkSRK-A*, *AkSRK-B* and *AkSRK-D—*are indeed associated with the female specificities of SI. Although associations with SI specificities for the other less frequent haplogroups—*AkSRK-C* and *AkSRK-E—*were not confirmed experimentally, they also exhibited very high similarities with known *SRK* sequences from *A. lyrata* and *A. halleri* (>98%), suggesting that specificities of SI are shared between species [59], [74].

Our investigation on nucleotide polymorphism and divergence, as well as the pattern of segregation in natural and experimental populations, allows us to address how these five *S*-haplogroups in allotetraploid *A. kamchatica* originated from their parental diploid species, *A. halleri* and *A. lyrata*. First, *AkSRK-A* and *AkSRK-C* of *A. kamchatica* are closer to the orthologs of *A. halleri* than to those of *A. lyrata*, and *AkSRK-D* and *AkSRK-E* of *A. kamchatica* are closer to the orthologs of *A. lyrata* than to those of *A. halleri* (Figure 2; Figure S1; Table S2). We also found that *AkSRK-B* in *A. kamchatica* showed 100% identity to *AhSRK-B* in *A. halleri*, although its ortholog of *A. lyrata* was not isolated in the present study. Second, the distributions of *AkSRK-A*, *AkSRK-B* and *AkSRK-C* were mutually exclusive, as were those of *AkSRK-D* and *AkSRK-E*, except for a minor presumable heterozygote of *AkSRK-B* and *AkSRK-C* (Figure 3; Figure 4). Third, the pattern of segregation in the F_2_ population significantly supports the model in which *AkSRK-A* and *AkSRK-B* are allelic while *AkSRK-D* is not allelic to them (Table 1). In addition, the pattern of polymorphism within a Kamchatka population is consistent with the model in which *AkSRK-B* and *AkSRK-C* are also allelic and segregating in a local population (Table S4). These independent lines of evidence suggest that *AkSRK-A, AkSRK-B* and *AkSRK-C* are *S*-alleles of the *A. halleri*-derived *S*-locus and that *AkSRK-D* and *AkSRK-E* are *S*-alleles of the *A. lyrata-*derived *S-*locus. Although we confirmed the association between *AkSRK-D* and the SI specificity of *A. halleri* in this study, the specificity is also likely to be shared with *A. lyrata* (discussed in [59], [61], [62]). While there are a few reports on the evolutionary history of the *S*-locus in cultivated allotetraploid species, particularly *Brassica napus* [79], [80], to our knowledge, this study is the first to demonstrate clearly how multiple *S*-haplogroups in a wild allotetraploid species originated from the parental species and spread throughout a geographically wide area.

### Pattern of nucleotide polymorphism and divergence of the *S*-locus

Shimizu-Inatsugi et al. [48] reported that multiple haplotypes of nuclear and chloroplast sequences were shared between allotetraploid *A. kamchatica* and its parental diploid species, suggesting independent origins of *A. kamchatica*. In particular, *A. kamchatica* and *A. halleri* share four identical chloroplast haplotypes, suggesting that at least four diploid individuals of *A. halleri* contributed independently to the multiple origins of *A. kamchatica* [48]. As three of the four suggested independent origins are manifested as distinct clusters of population structure, independent origins combined with range expansion out of Asia appear to affect the population structure of *A. kamchatica* profoundly [48]. A comparison between the geographic distribution of *S*-haplogroups and the population structure inferred from other loci thus illustrates how independent origins of *A. kamchatica* contributed to form the current pattern of polymorphism of two *S*-loci: the *A. halleri*-derived *S-*locus and the *A. lyrata*-derived *S-*locus. We found that the distribution of three *A. halleri*-derived *S*-haplogroups was significantly correlated with population structure. Given that the population structure of *A. kamchatica* is profoundly affected by its multiple independent origins, the concordance between population structure and the distribution of *A. halleri*-derived *S*-haplogroups suggests that the composition of the gene pool of the *A. halleri*-derived *S-*locus would be explained at least partially by the independent origins of this species. In contrast, there was no significant correlation found for the *A. lyrata*-derived *S-*locus, on which *AkSRK-D* is markedly more frequent than *AkSRK-E.* A similar pattern was observed in the European population of *A. thaliana*, in which the haplogroup A was much more frequent than the haplogroup C and thus the pattern of polymorphism at the *S*-locus showed deviation from the population structure [41]. A coalescent approach based on *SRK* sequences of species-wide samples as well as genome-wide polymorphism data would serve to unveil more precisely how the five *S*-haplogroups originated in *A. kamchatica*, especially by quantifying the effects of random genetic drift, population expansion out of Asia, and independent origins of species, respectively.

Such a population genomic approach should also reveal the demographic history during the loss of SI in *A. kamchatica*. The retained functionality of the female SI components including the downstream signaling pathway shown in this study implies a fairly recent loss of SI in *A. kamchatica*. Indeed, estimates of the average divergence time of the nuclear loci (*WER* and *CHS*) are less than 250 thousand years (kyrs), although this divergence time may not necessarily correspond to the timing of the loss of SI (Table S6). Dating of the loss of SI has also been estimated in other self-compatible lineages, *A. thaliana* (<314 kyrs [62]; <500 kyrs [81]), *C. rubella* (∼27 kyrs [43]) and *L. alabamica* (∼150 kyrs and 12–48 kyrs in two independent lineages [44]), assuming the substitution rate published by Koch et al. [75]. These timings of evolutionary transitions are generally concordant with the recent glacial–interglacial period that greatly influenced the distribution of many plant and animal species [82]. Indeed, recent genetic bottlenecks and/or population expansions have been suggested for *A. thaliana* [83]–[85], *C. rubella* [43], [73], *L. alabamica* [44] and in selfing populations of *A. lyrata* in North America [86]–[88]. The low nucleotide diversity of *A. kamchatica* in comparison with its parental species is also consistent with a bottleneck during polyploidization (0.0006–0.0012 in *A. lyrata*-derived homeologs of *A. kamchatica* and 0.0008–0.0026 in *A. halleri*-derived homeologs of *A. kamchatica*, versus 0.0150 in *A. halleri*, 0.0230 in *A. lyrata* subsp. *petraea* and 0.0031 in *A. lyrata* subsp. *lyrata* [48], [89]).

As range expansions could be accompanied by reduced mate availability and increased pollen limitation [7], [90]–[92], reproductive assurance might have played a role in these examples of the loss of SI. Particularly, mates with the same ploidy level would be limited after polyploidization in *A. kamchatica* [1], [66]–[70]. Another possibility has been suggested by recent theoretical and simulation studies, which proposed that range expansion might promote the evolution of selfing [93]. This is because inbreeding depression would be reduced in marginal populations where deleterious mutations are fixed or purged by strong genetic bottlenecks [93]–[95]. If inbreeding depression falls below a certain threshold, disrupted *S*-haplogroups causing self-compatibility are expected to rapidly replace all functional *S*-haplogroups via an inherent transmission advantage [24], [27]. Although this is not mutually exclusive to reproductive assurance [44], [96], further analyses on the demographic history and the dating of the loss of SI should contribute to our ability to address the relative importance of transmission advantage and reproductive assurance for the evolution of self-compatibility during range expansions.

### Dominance interactions among *S*-haplogroups and the loss of SI

The molecular mechanism underlying the loss of SI in polyploid species is still not well understood, although the relationship between polyploidy and self-compatibility has been debated for more than 60 years [1], [65], [66]. Whereas polyploidization is known to disrupt SI almost invariably in gametophytic SI systems where specificities are determined by haploid genotypes of gametes [97], [98], in sporophytic SI systems where specificities are determined sporophytically by the diploid parental genotypes, polyploidization in itself does not necessarily induce the loss of SI (e.g., [57]). We speculate that, in sporophytic SI systems such as those of the Brassicaceae, dominance interactions among *S*-haplogroups might have played an important role in the loss of SI in polyploid species, as is also implied in a study of synthetic interspecific hybrids by Nasrallah et al. [99]. In the Brassicaceae, complex dominance interactions among *S*-haplogroups have been reported [52], [55], [56], [60], [100]–[103]. Given that the female components are functionally intact in *A. kamchatica*, as shown by our interspecific crosses, both homeologous copies of the *SCR* gene ought to have lost their function. However, gene-disruptive mutations at both of the homeologous *SCR* genes might not necessarily be required if the dominant *SCR* harboring a gene-disruptive mutation suppresses the expression of the recessive *SCR* [80], [104]. Thus, dominance interactions could facilitate the evolution of self-compatibility by a single mutation in polyploid species. This is indeed suggested for *Brassica napus*, which is also an allotetraploid species that originated from hybridization of *B. rapa* and *B. oleracea* [80]. In a cultivar of *B. napus*, neither of the homeologous *SP11/SCR* genes is expressed. In artificially synthesized *B. napus* lines, *SP11/SCR* alleles from *B. rapa* showed dominance over *SP11/SCR* alleles from *B. oleracea*, suggesting that the nonfunctional dominant *SP11/SCR* allele suppressed the expression of the recessive *SP11/SCR* allele [80].

In *A. lyrata* and *A. halleri*, such dominance relationships between *S*-haplogroups have been characterized. Thus, the *S*-haplogroup of *S*_12_ in *A. halleri*, which is orthologous to haplogroup D of *A. kamchatica*, has been suggested to belong to a relatively dominant class [60]. Given that the dominance relationship is consistent between *A. halleri* and *A. kamchatica*, haplogroup D would also be dominant in *A. kamchatica* and might have suppressed the expression of its homeologous counterparts, such as haplogroup C, which is orthologous to the most recessive *S*-haplogroup of *S*_1_ in *A. lyrata* and *A. halleri* [52], [60], [61], [103]. While we showed that haplogroups B and D are codominant in pistils in *A. kamchatica* (Figure 6), their dominance rank might be different in pollen, as dominance is known to occur more frequently in pollen than in pistils [60].

It is worth noting that haplogroup C, which is orthologous to the most recessive *S*-haplogroup of *S*_1_ in *A. lyrata* and *A. halleri*, was found at a relatively low frequency in *A. kamchatica* (5.1%). In contrast, *S*_1_ shows the highest frequencies in self-incompatible populations of *A. lyrata* (33%; [55]) and *A. halleri* (26.3%; [60]), because recessive haplogroups are hidden from negative frequency-dependent selection in heterozygotes, whereas the most dominant *S*-haplogroups are always exposed to selection [60], [105], [106]. While random genetic drift due to population bottlenecks could explain this contrasting change in frequency, we hypothesize that dominant haplogroups are more likely to be found in self-compatible populations. This is because a dominant haplogroup with gene-disruptive mutations should repress the expression of another specificity, and thus spread more rapidly than a recessive self-compatible mutation [42]. Consistent with this hypothesis, a nearly fixed haplogroup in *A. thaliana* has been shown experimentally to be a dominant allele in *A. halleri* [42], [60].

The molecular basis of the dominance relationship has recently been unveiled in *Brassica*, where the expression of the recessive haplogroups is specifically silenced by methylation of promoter regions induced by *trans*-acting small noncoding RNAs originated from dominant haplogroups [101], [107], [108]. Investigation of the dominance relationships among *S*-haplogroups found in *A. kamchatica*, the pattern of expression of the *SCR* genes and their methylation status in *A. kamchatica* will allow us to understand how dominance interactions might have contributed to the loss of SI among polyploid species.

### Mutations in pollen components were responsible for the loss of SI

The retained functionality of the female SI components shown by the sequence analysis of *SRK* and interspecific crosses suggests that the degradation of male components, possibly the *SCR* gene, was responsible for the loss of SI in *A. kamchatica*. We also suggest that the gene-disruptive mutations found in the *SRK* gene of some accessions are not primary mutations causing the loss of SI but rather represent secondary and independent decay. Thus, these would represent mutations that have accumulated after the evolution of self-compatibility, although pleiotropy of *SRK* might have played a role in maintaining its functionality and slowed this process [42], [45].

Interestingly, evolutionary models predict that mutations disabling the male specificity of the SI system are expected to be more strongly selected than mutations disabling female specificity [25], [28]. This is because these disabled pollen grains are transmitted as outcrossing pollen to offspring with a higher probability than other wild-type (functional) pollen. In contrast, mutations disabling the female specificity are not likely to benefit from the increased fitness over other functional pistils, as long as pollinators frequently visit and supply pollen grains that belong to various specificities [109]. Our finding in *A. kamchatica* is thus consistent with the prediction of these models.

Mutations driving the loss of SI in the male component have also been reported in *A. thaliana* [41]. Similarly, the loss of SI in *C. rubella* might have been driven by a gene-disruptive mutation in the male component, because *SRK* appears to retain the full-length coding region in some accessions, indicating that mutations at *SRK* are unlikely to have been the primary cause for the loss of SI ([43]; reviewed in [110]). Busch et al. [44] also reported that self-compatibility in *L. alabamica* might have originated by the loss of function of the male component, possibly *SCR*. These recent extensive studies in wild Brassicaceae species demonstrate that the evolution of self-compatibility tends to be driven by mutations in the male rather than in the female components (Table 2).

**Table 2:** Numbers of examples in which the primary mutations involved in the loss of SI are attributable to male or female components of self-incompatibility.

	Male component	Female component
Wild populations†	4	0
Cultivated populations†	1	5

The pattern is significantly different between wild and cultivated populations (two-tailed Fisher's exact test *p* = 0.0476; one-tailed *p* = 0.0238).

**†:** Wild populations: *Arabidopsis thaliana* [41], *A. kamchatica* (this study), *Capsella rubella* [43] and *Leavenworthia alabamica* [44]. Cultivated populations: *Brassica napus* [79], [80], *B. oleracea* [111] and *B. rapa* [112]. See Table S8 for details of mutations involved in the loss of SI in cultivated *Brassica* populations.

In contrast, in cultivated *Brassica*, extensive functional analyses have identified gene-disruptive mutations both in male and in female components, and the pattern is significantly different from that observed in wild populations (Table 2; Table S8; two-tailed Fisher's exact test *p* = 0.0476; one-tailed *p* = 0.0238; [79], [80], [111], [112]). This contrasting pattern suggests that the male-skewed frequency of mutations found in recent studies of wild populations could be attributed to the process of natural selection and spread in wild populations [25], [28], rather than to the mechanistic natures of mutations, such as differences in mutation rate between male and female components. If such mechanistic reasons were important, similar patterns would have been observed in both wild and cultivated populations. Indeed, the context of selective pressure would be very different between wild and cultivated populations. In a wild population, as mentioned above, mutations disabling SI would be selected in terms of the transmission advantage, i.e., number of compatible mates in a population [25], [28]. In cultivated populations, these gene-disruptive mutations would be positively selected by humans based on the self-compatible phenotype *per se*. Moreover, because the female *SRK* gene is about 10 times longer than the male *SCR*, it may decay even faster than the male component during domestication, as indeed shown in cultivated *Brassica* (Table 2; Table S8).

### Conclusion and future perspectives

Our combination of population genetic analyses and crossing experiments suggests that degradation of the male components was primarily responsible for the loss of SI in *A. kamchatica*. As we have demonstrated in the present study, functional confirmations, such as crossings between individuals sharing the same SI specificity, will further corroborate the genetic basis for the loss of SI in other selfing species. First, sequence analysis alone cannot provide definitive evidence of gene function. For example, in previous studies of the evolution of self-compatibility in *A. thaliana*, a splicing mutation in the female gene *SRK-B* was found in the Cvi-0 accession while the male gene *SCR-B* did not have any obvious gene-disruptive mutations [38]. However, transgenic experiments suggested that *SCR-B* was also nonfunctional, possibly due to some amino acid changes, so it is not clear whether the primary mutation occurred in male or female components [40]. Second, functional analyses may reveal multiple origins of self-compatibility within species and contribute to an increase in the number of empirical examples, although in the present study, which took a conservative approach, each wild species was counted as one example (Table 2). While more empirical examples in various species should be gathered to better understand the general pattern of mutations in the evolution of self-compatibility, functional confirmations and analyses of the pattern of polymorphisms are both essential to disentangle mutational histories in the loss of SI.

## Materials and Methods

### Plant materials

*Arabidopsis kamchatica* consists of two subspecies, *A. kamchatica* subsp. *kamchatica* and *A. kamchatica* subsp. *kawasakiana* [46]. *A. kamchatica* subsp. *kamchatica* is a perennial, described originally from Kamchatka, Russia. It is also reported from East Asia (Far East Russia, China, Korea, Japan and Taiwan) and North America (Alaska, Canada and the Pacific Northwest of the USA). The second subspecies, *A. kamchatica* subsp. *kawasakiana*, is an annual found in sandy open habitats along seashores or lakeshores in lowlands in western Japan. Tetraploid chromosome number counts (2*n* = 32 and *n* = 16_II_) were reported from samples in Japan, Far East Russia, Alaska and Canada, representing both subspecies 46,48.

Altogether, 48 populations of *A. kamchatica* were sampled (43 from *A. kamchatica* subsp. *kamchatica* and five from *A. kamchatica* subsp. *kawasakiana*), including one to three individuals per population, giving a total of 60 accessions (Table S1). The sample locations included Far East Asia (Taiwan and Japan), Far East Russia (Kamchatka Peninsula and Okhotsk), Alaska, Canada and the northwest of the USA (Washington), covering the majority of the distribution range of both subspecies. In addition, five accessions of *A. halleri* were used for interspecific crosses with *A. kamchatica* and for obtaining full-length sequences of *SRK* (Table S1; see also the section “Interspecific crosses between *A. halleri* and *A. kamchatica*” for details).

### General methods for isolation of genomic/complementary DNA, genotyping, sequencing, and construction of phylogenetic trees

Genomic DNA was isolated from young leaves using Plant DNeasy Mini kits (Qiagen, Hilden, Germany). Total RNA was extracted from floral buds and flower tissues with RNeasy kits (Qiagen). The 5′ and 3′ ends of complementary DNA (cDNA) sequences were isolated by 5′ and 3′ Rapid Amplification of cDNA Ends (RACE) with the 5′/3′ 2nd Generation RACE Kit (Roche Applied Science, Indianapolis, IN, USA). PCR was performed with ExTaq (TaKaRa Bio Inc., Shiga, Japan) or iProof High Fidelity DNA Polymerase (Bio-Rad, Hercules, CA, USA). Primers used for amplification and genotyping are shown in Table S3, with the respective annealing temperatures and elongation times. Genotyping was based on the presence or absence of PCR products. Direct DNA sequencing was conducted at the Institute of Plant Biology, University of Zurich, using a PRISM 3730 48-capillary automated sequencer (Applied Biosystems, Foster City, CA, USA). Sequence assemblies and alignments were performed in CodonCode Aligner 3.7.1 (CodonCode, Dedham, MA, USA) and BioEdit v 7.0.5 [113]. Minor adjustments to optimize the alignments were made by eye. Sequence data have been deposited in GenBank under accession numbers JX114752–JX114778. MEGA 5 was used for the construction of a phylogenetic tree [114]. Phylogenetic trees were obtained using the neighbor-joining method on pairwise proportions of nucleotide divergence. The evolutionary distances were computed using the Kimura two-parameter method.

### PCR–based screening and sequencing of the entire coding region of *SRK*

To survey the *S*-haplogroups in *A. kamchatica* across its distribution range, we performed a PCR-based screening of the *SRK* gene using two kinds of primer sets: (1) “general primers”, designed in conserved regions of the *S*-domain of *SRK* and known to amplify *SRK* sequences that belong to a number of haplogroups (“Aly13F1” and “SLGR” [52], [55]); and (2) “haplogroup-specific primers”, designed in polymorphic regions of the *S*-domain of *SRK* [55]. Based on the obtained sequences, new haplogroup-specific primers were designed for each haplogroup (Table S3). Although we found sequences in several accessions that were highly similar to the *Aly13-7* sequence, Mable et al. [55] reported that *Aly13-7* is not associated with SI. Thus, these sequences were excluded from further analyses.

To investigate the functionality of *SRK*, the entire coding sequences of *SRK* were obtained by RACE PCR using the haplogroup-specific primers designed in the *S*-domain of *SRK*. The primers used for RACE PCR are listed in Table S3.

### Bayesian clustering for the inference of population structures

To examine the associations between the geographic distribution of the *S*-haplogroups and the population structure (see the next section for details), we reexamined the population structure of *A. kamchatica*, which was originally inferred by Shimizu-Inatsugi et al. [48], by adding the Okhotsk population that was not included in the former study. From two accessions of this population, we obtained nucleotide sequences of the genes *WER* (*WEREWOLF*) and *CHS* (*CHALCONE SYNTHASE*). By including these data in the dataset of Shimizu-Inatsugi et al. [48], population structure was inferred by the Bayesian clustering algorithm implemented in the InStruct program [115]. The programs CLUMPP [116], DISTRUCT [117] and *ΔK* statistic [118] were used to summarize and interpret the outputs. The parameters used were the same as in Shimizu-Inatsugi et al. [48], except for the number of independent runs: 10 instead of 20. In addition to the software InStruct, we also used the software STRUCTURE 2.2.3, which is not able to consider inbreeding explicitly [119]. (See Text S1 and Figure S5 for details.)

### Association analysis of *SRK* genotypes and population structures

To examine associations between the geographic distribution of the *S*-haplogroups and the population structure inferred from polymorphisms of other nuclear loci (*WER* and *CHS*), Cramer's coefficient *V* (also called *φ*) was calculated for two sets of *SRK* (*A. halleri*-derived *AkSRK-A*, *AkSRK-B* and *AkSRK-C* and *A. lyrata*-derived *AkSRK-D* and *AkSRK-E*). Cramer's coefficient *V* measures the strength of association or interdependence between two categorical variables [120], [121]. Its statistical significance was assessed using Fisher's exact test. (See Table S9 for cluster assignments based on *K* = 2, 3, or 4.) Heterozygotes or accessions that did not bear corresponding *SRK* were excluded from the analysis. All statistical analyses were conducted with R 2.10.0 [122].

### Calculation of nucleotide divergence of *SRK*

To obtain insight into which parental species *SRK* of *A. kamchatica* were derived from, we calculated mean values of *K*, *K*s (the proportion of synonymous substitutions per synonymous site) and *K*a (the proportion of nonsynonymous substitutions per nonsynonymous site) from the outgroups *A. halleri* and *A. lyrata* for all *SRK* sequences obtained in *A. kamchatica*, using MEGA 5 [114]. We used all the sequences of *A. kamchatica* and *A. halleri* obtained in this study (see Table S1 for which accessions were used). In addition, we used the following publicly available sequences for the calculation: *AlSRK22* [52], *AhSRK26* [59], *AlSRK01* [61], *AhSRK01* [61], *AlSRK42* [59], *AhSRK12* [58], *AlSRK17* [54] and *AhSRK02* [58]. Specifically, we calculated the nucleotide divergence of the following sequence pairs: (1) *AkSRK-A* from *AlSRK22* and from *AhSRK26*; (2) *AkSRK-B* from *AhSRK-B*; (3) *AkSRK-C* from *AlSRK01* and from *AhSRK01*; (4) *AkSRK-D* from *AlSRK42* and from *AhSRK12*; and (5) *AkSRK-E* from *AlSRK17* and from *AhSRK02*.

### Estimation of divergence time from two parental species

To understand the approximate timescale of the evolutionary loss of SI in *A. kamchatica*, the divergence time of *A. kamchatica* from two parental species was calculated based on four nuclear loci (*CHS*-lyr, *CHS*-hal, *WER*-lyr and *WER*-hal). We first calculated the nucleotide divergence on these loci using publicly available data [48] as well as newly obtained sequence data (see the section “Bayesian clustering for the inference of population structures”). For *A. lyrata*, two accessions from Far East Russia were reported to show the highest nucleotide similarities with *A. lyrata*-derived homeologs of *A. kamchatica* among those surveyed by Shimizu-Inatsugi et al. [48], suggesting that they are closest to one of the founding parents of *A. kamchatica*. In contrast, other individuals of *A. lyrata* from Europe and North America were not as close to the *lyrata*-derived homeologs of *A. kamchatica*. Therefore, we calculated the divergence of *A. kamchatica* from these two *A. lyrata* accessions from Far East Russia, as this would better represent the split from the parental species. Standard errors and 95% confidence intervals were calculated for all estimates. All estimates were corrected using the Jukes–Cantor method [123]. As two clusters were suggested in the InStruct analysis (Figure 3), we also calculated the clusterwise nucleotide divergence from *A. halleri* and *A. lyrata* (Table S10.). (See the above section, “Association analysis of *SRK* genotypes and population structures”, for the cluster assignment.)

Based on the calculated nucleotide divergence, we estimated the divergence time between *A. kamchatica* and the two parental species *A. lyrata* and *A. halleri*. The estimation was based on the expression *T* = *K*/2*r*, where *T* is the time to the most recent common ancestor, *K* is the nucleotide divergence and *r* is the substitution rate [124]–[126]. Note that our estimation of divergence time assumes a constant rate of evolution throughout the tree [127]. Here we employed two estimates of mutation rates: the synonymous mutation rate of 1.5×10^−8^±0.5×10^−8^ per site per year [75], which is commonly used in studies of the evolution of self-compatibility [44], [62], and a mutation rate of 7.1×10^−9^±0.7×10^−9^ per site per generation, which was estimated using mutation accumulation lines [76]. While we assumed a generation time of two years [128], we note that this could cause an overestimation or an underestimation, because some accessions of *A. kamchatica* are reported to be annual plants [47], [48] and because an effect of seed dormancy was not considered, respectively. Note that estimates based on the mutation rate given by Koch et al. (2000) are independent from the generation time, because its unit is base pair per year, not per generation. For calculating the 95% upper and lower bounds of divergence time, the 95% upper and lower bounds of nucleotide divergence and the 95% lower and upper bounds of mutation rates were used, respectively. For estimating the divergence time, we used the nucleotide divergence values of *CHS*-hal and *WER*-hal from corresponding orthologs in *A. halleri*, and those of *CHS*-lyr and *WER*-lyr from corresponding orthologs in *A. lyrata* from Far East Russia.

### Interspecific crosses between *A. halleri* and *A. kamchatica*

We conducted interspecific crosses between *A. halleri* and *A. kamchatica* to test whether the full-length *SRK* sequences and other components involved in the female signaling pathway are functional, and also to test whether the male components are not functional in *A. kamchatica*. To screen *A. halleri* plants bearing haplogroups A, B or D, genotypes of *AkSRK* were surveyed in *A. halleri* from Mt Ibuki (35.42°N, 136.40°E), Ohara (35.16°N, 135.84°E) and Tada-Ginzan (34.90°N, 135.35°E) in Japan. We used them as both pollen and pistil donors for the crossing experiments, because individuals that bear *SRK-A*, *SRK-B* or *SRK-D* should bear the haplogroup A, B or D of the *S*-locus (encompassing *SCR-A*, *SCR-B* or *SCR-D*), respectively, given the tight linkage between *SRK* and *SCR*. Three *A. halleri* plants were used for each haplogroup. The haplogroup-specific primers listed in Table S3 were used for this screening. Two *A. halleri* plants from Boden and Beride in Switzerland, which bear neither *SRK-A*, *SRK-B*, nor *SRK-D*, were also used as pollen donors (Table S1). To confirm the *SRK* genotypes, eight *A. kamchatica* accessions were used for the crossing experiments (Table S1). Three of these eight accessions are reported to be capable of selfing [48], and the other five accessions were confirmed in this study (data not shown). Plants used in the pollination assay were grown at 22°C under a 16 h light/8 h dark cycle. We removed anthers from flower buds and carefully confirmed that stigmas were not contaminated by self-pollen using a stereomicroscope. Flowers were harvested 2–3 h after pollination when *A. kamchatica* was the pistil donor, or 24 h after pollination when *A. halleri* was the pistil donor. Harvested flowers were fixed in a 9∶1 mixture of ethanol and acetic acid, softened for 10 min in 1 M NaOH at 60°C and stained with aniline blue in a 2% K_3_PO_4_ solution. Pistils were mounted on slides to examine the pollen tubes using epifluorescence microscopy [41], [129]. In compatible crosses, more than 100 pollen tubes typically penetrate the stigma and penetration of <20 pollen tubes was considered as a criterion of incompatible crosses, following the common criteria of previous work in *Arabidopsis* [31], [41]. The results did not change even if a more stringent criterion of <10 pollen tubes was used. Although pollen tube growth was observed in most of the crosses to evaluate incompatible and compatible reactions, in a few combinations of crosses where *A. halleri* was the pistil donor, we alternatively used the lengths of siliques as a criterion of incompatible crosses (see Figure S4). A silique length of <5 mm was considered to be the criterion of an incompatible cross.

### F_2_ generation segregation analysis

To confirm the disomic inheritance and the allelic relationship of *AkSRK-A* and *AkSRK-B*, F_2_ segregation analysis was conducted using *A. kamchatica*. F_1_ plants were generated using the Biwako accession from Japan as the pistil donor and the Potter accession from Alaska as the pollen donor. (See Table S1 for the detailed geographic locations of these accessions.) Ninety-five F_2_ plants were generated by selfing of two F_1_ plants and genotyped using haplogroup-specific primers for *AkSRK-A*, *AkSRK-B* and *AkSRK-D* (Table S3). While *AkSRK-A* and *AkSRK-D* were amplified in the Biwako accession and *AkSRK-B* and *AkSRK-D* were amplified in the Potter accessions (see Table S1 for details), all of *AkSRK-A*, *AkSRK-B* and *AkSRK-D* were amplified in F_1_ plants. Using χ^2^ tests with R 2.10.0 [122], the goodness-of-fit for each of the following inheritance models was calculated: (1) disomic and allelic, (2) disomic and nonallelic, (3) tetrasomic and allelic and (4) tetrasomic and nonallelic. The expected frequencies of segregants are described in Table 1.

## Supporting Information

Figure S1 Phylogenetic trees of *SRK* sequences from haplogroups A (A), B (B), and C (C) from *A. halleri*, *A. lyrata* and *A. kamchatica*. This phylogeny was obtained by the neighbor-joining method on pairwise proportions of nucleotide divergence. In total, 552 (A), 567 (B) and 449 (C) nucleotide positions were used. The evolutionary distances were computed using the Kimura two-parameter method. See Table S10 for accession numbers of these sequences deposited in GenBank. *SRK* sequences obtained in this study are shown in red.
(TIF)

Figure S2 Results of population clustering based on the data for the nuclear *WER* and *CHS* genes using InStruct software. (A) Mean posterior probability of the data ln *P*(*X*|*K*) over 10 runs for each *K*-value. (B) Plot of *ΔK* for each *K*.
(TIF)

Figure S3 Alignment of the predicted amino acid sequences of *AkSRK-A*, *AkSRK-B*, *AkSRK-C*, *AkSRK-D* and *AkSRK-E*, deduced from their DNA sequences. A 1-bp deletion causing a frameshift mutation in *AkSRK-C* is shown as a red “X” (position 67) and subsequent amino acids are shown as if the frameshift did not happen. AkSRK-D* denotes *AkSRK-D* from Murodo bearing a deletion of 15 amino acids caused by a 45-bp deletion in *AkSRK-D* genomic DNA. The site of an approximate 1,700-bp insertion in *AkSRK-A* of the Biwako accession, leading to a premature stop mutation, is indicated in blue (between positions 751 and 752). Subsequent amino acids are shown as if the insertion did not happen. Twelve conserved cysteine residues are indicated in orange [77], [78]. Asterisks denote stop codons.
(TIF)

Figure S4 Interspecific crosses between *A. halleri* (pistil donor) and *A. kamchatica* (pollen donor), and control crosses within *A. halleri.* Unless indicated by asterisks, numerators denote crosses where more than 20 pollen tubes penetrated the stigma (compatible crosses). If indicated, numerators denote crosses where the length of siliques was >5 mm (see Methods). Denominators denote the total number of crosses conducted in each combination.
(TIF)

Figure S5 Results of population clustering based on the data of cpDNA and nuclear *WER* and *CHS* genes using STRUCTURE software. See the caption for Figure S1 for details. (A) Inference of population structure for the clustering of *K* = 2, 3, and 4. (B) Mean posterior probability of the data ln *P*(*X*|*K*) over 10 runs for each *K*-value. (C) Plot of *ΔK* for each *K*.
(TIF)

Table S1 Geographic locations of materials used in this study, list of accessions used for each analysis, and the results of PCR-based genotyping.
(XLS)

Table S2 Nucleotide divergence of *SRK* from two parental species, *A. halleri* and *A. lyrata*.
(XLS)

Table S3 Primer list.
(XLS)

Table S4 Pattern of intrapopulation segregation in a Kamchatka population from Petropavlovsk Kamchatskii, Mishenaya gora.
(DOC)

Table S5 Nucleotide divergence of the *CHS* and *WER* genes from the two parental species *A. halleri* and *A. lyrata*.
(XLS)

Table S6 Estimated divergence time in years from parental species under two estimates of the mutation rate: Koch et al. [75] and Ossowski et al. [76].
(XLS)

Table S7 Intraspecific crosses between two accessions to exclude the possibility that the male components of haplogroup D of the Murodo accession and of haplogroup A of the Biwako accession remain functional.
(DOC)

Table S8 Summary of mutations suggested to be responsible for the loss of SI in cultivated *Brassica* populations.
(XLS)

Table S9 Cluster assignments of each accession based on the analyses by InStruct and STRUCTURE, given the number of clusters *K* = 2, 3 or 4. The geographic location of each accession is also shown.
(XLS)

Table S10 GenBank accession numbers of sequences used to generate phylogenetic trees (Figure 1; Figure S1)
(XLS)

Text S1 Population Structure Based on the Software STRUCTURE.
(DOC)
