# Supplementary figures and images for: Recent Loss of Self-Incompatibility by Degradation of the Male Component in Allotetraploid Arabidopsis kamchatica
Source: PLoS Genet. 2012 Jul 26;8(7):e1002838. doi: 10.1371/journal.pgen.1002838 (PMC3405996; doi:10.1371/journal.pgen.1002838)

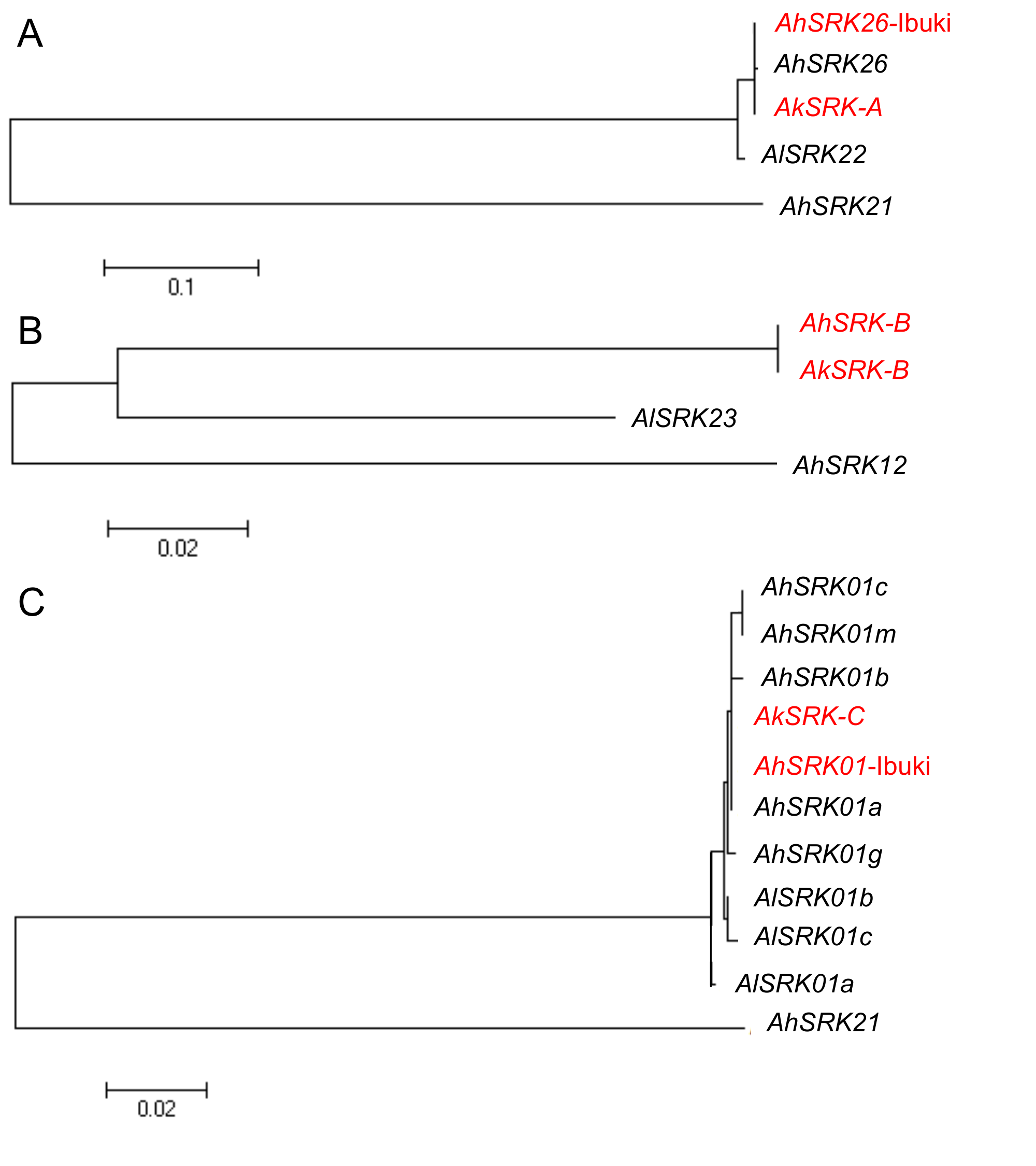

Supplement: Figure S1 — Phylogenetic trees of SRK sequences from haplogroups A (A), B (B), and C (C) from A. halleri, A. lyrata and A. kamchatica. This phylogeny was obtained by the neighbor-joining method on pairwise proportions of nucleotide divergence. In total, 552 (A), 567 (B) and 449 (C) nucleotide positions were used. The evolutionary distances were computed using the Kimura two-parameter method. See Table S10 for accession numbers of these sequences deposited in GenBank. SRK sequences obtained in this study are shown in red. (TIF) [file pgen.1002838.s001.tif]

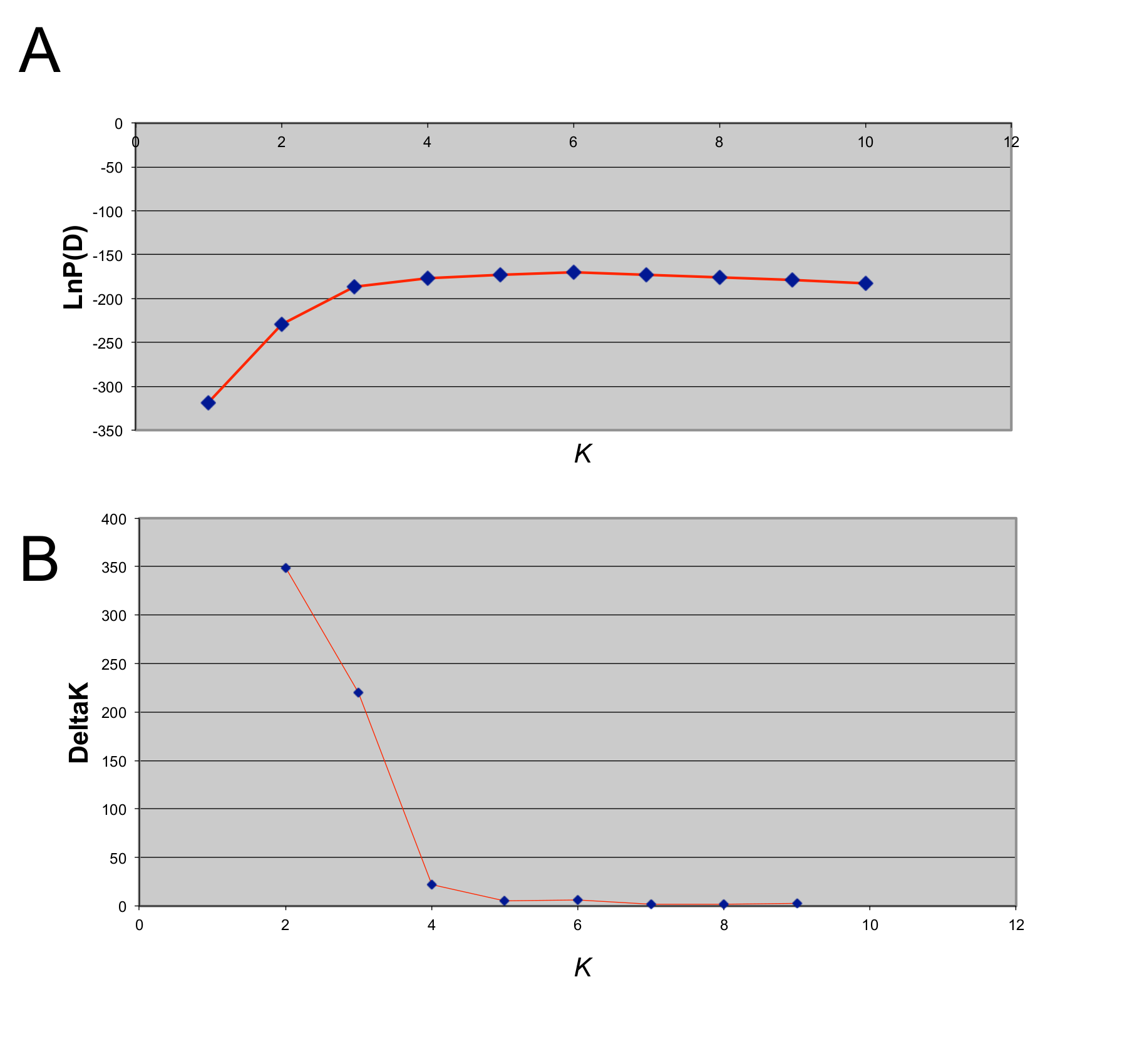

Supplement: Figure S2 — Results of population clustering based on the data for the nuclear WER and CHS genes using InStruct software. (A) Mean posterior probability of the data ln P(X|K) over 10 runs for each K-value. (B) Plot of ΔK for each K. (TIF) [file pgen.1002838.s002.tif]

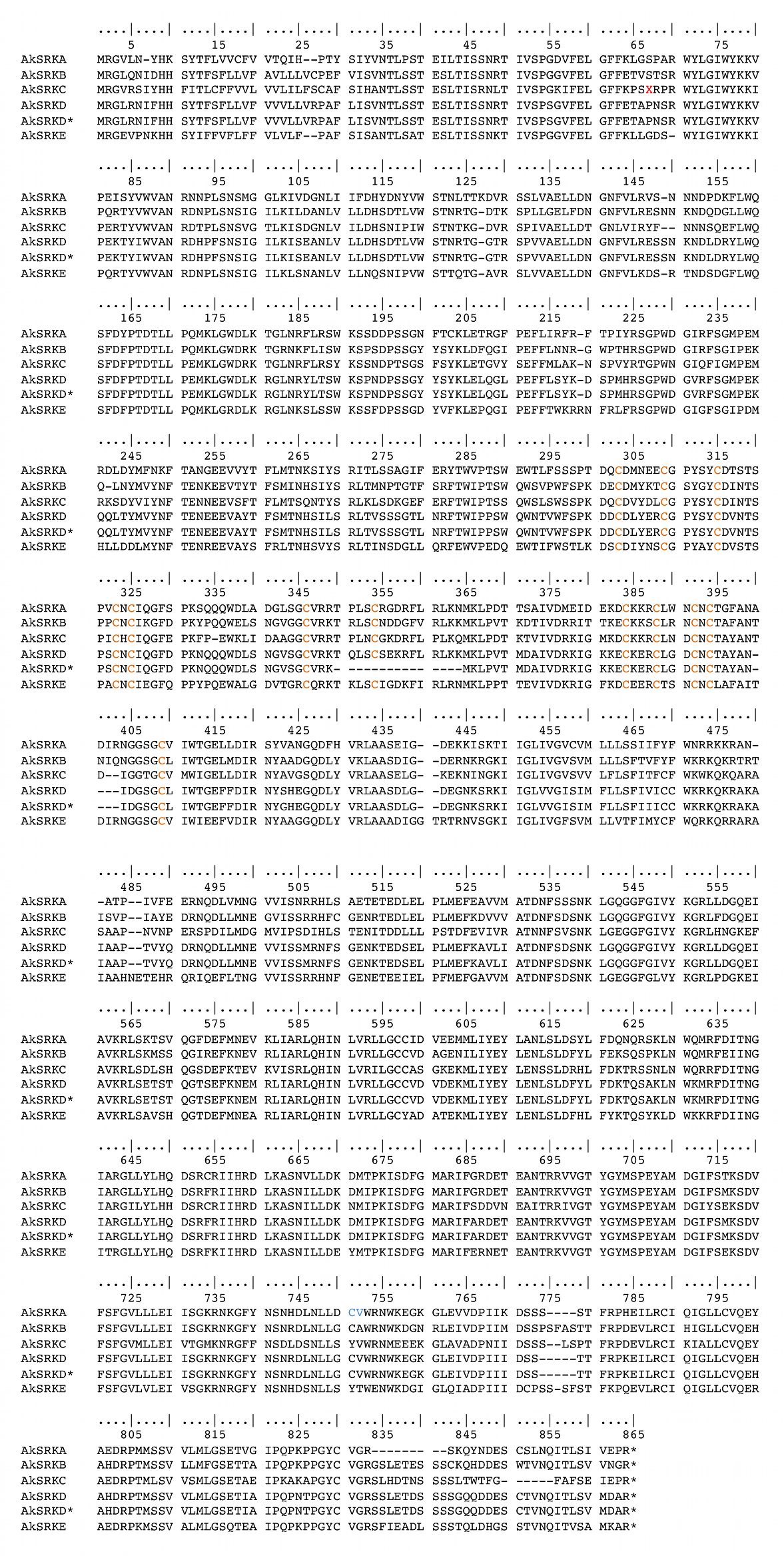

Supplement: Figure S3 — Alignment of the predicted amino acid sequences of AkSRK-A, AkSRK-B, AkSRK-C, AkSRK-D and AkSRK-E, deduced from their DNA sequences. A 1-bp deletion causing a frameshift mutation in AkSRK-C is shown as a red “X” (position 67) and subsequent amino acids are shown as if the frameshift did not happen. AkSRK-D* denotes AkSRK-D from Murodo bearing a deletion of 15 amino acids caused by a 45-bp deletion in AkSRK-D genomic DNA. The site of an approximate 1,700-bp insertion in AkSRK-A of the Biwako accession, leading to a premature stop mutation, is indicated in blue (between positions 751 and 752). Subsequent amino acids are shown as if the insertion did not happen. Twelve conserved cysteine residues are indicated in orange [77], [78]. Asterisks denote stop codons. (TIF) [file pgen.1002838.s003.tif]

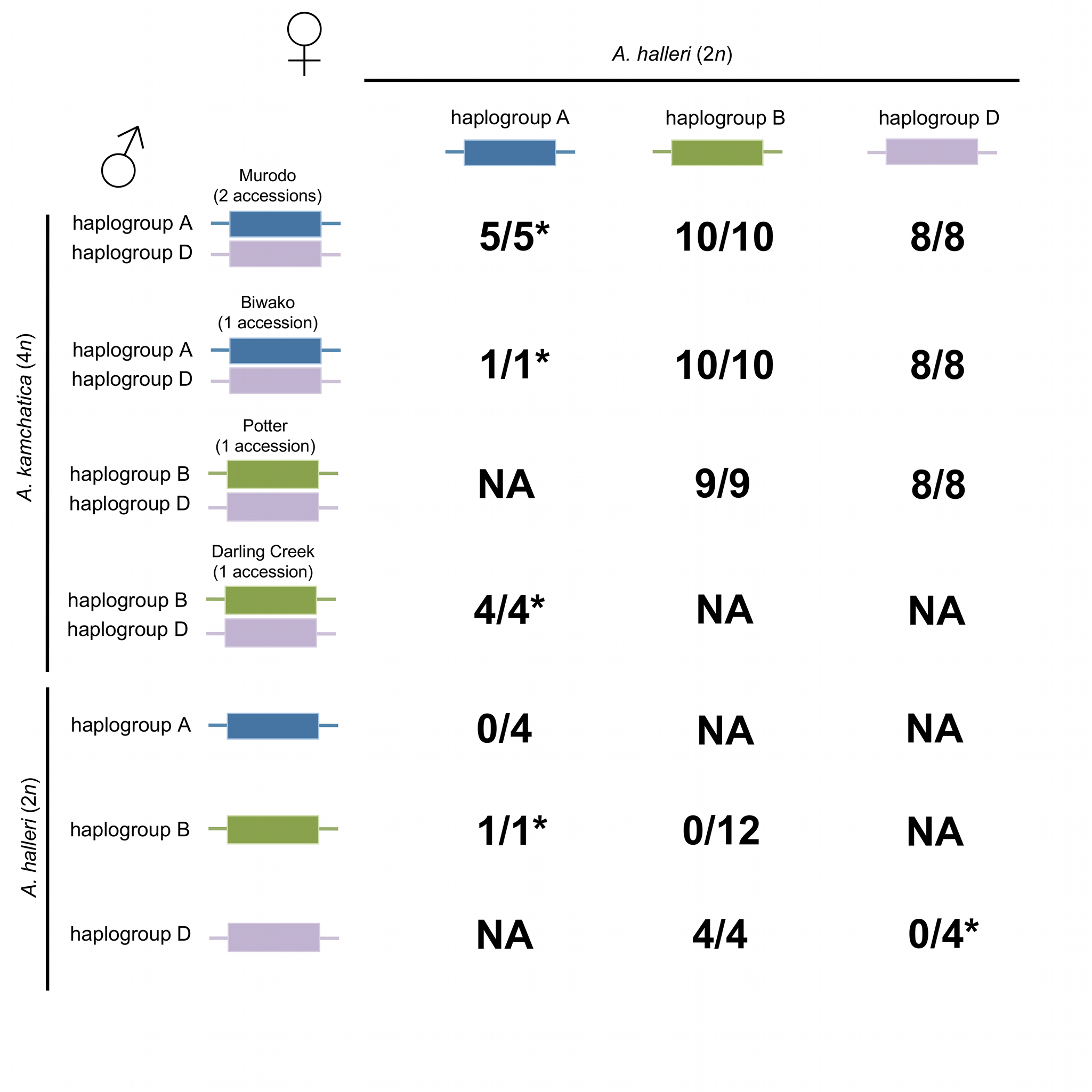

Supplement: Figure S4 — Interspecific crosses between A. halleri (pistil donor) and A. kamchatica (pollen donor), and control crosses within A. halleri. Unless indicated by asterisks, numerators denote crosses where more than 20 pollen tubes penetrated the stigma (compatible crosses). If indicated, numerators denote crosses where the length of siliques was >5 mm (see Methods). Denominators denote the total number of crosses conducted in each combination. (TIF) [file pgen.1002838.s004.tif]

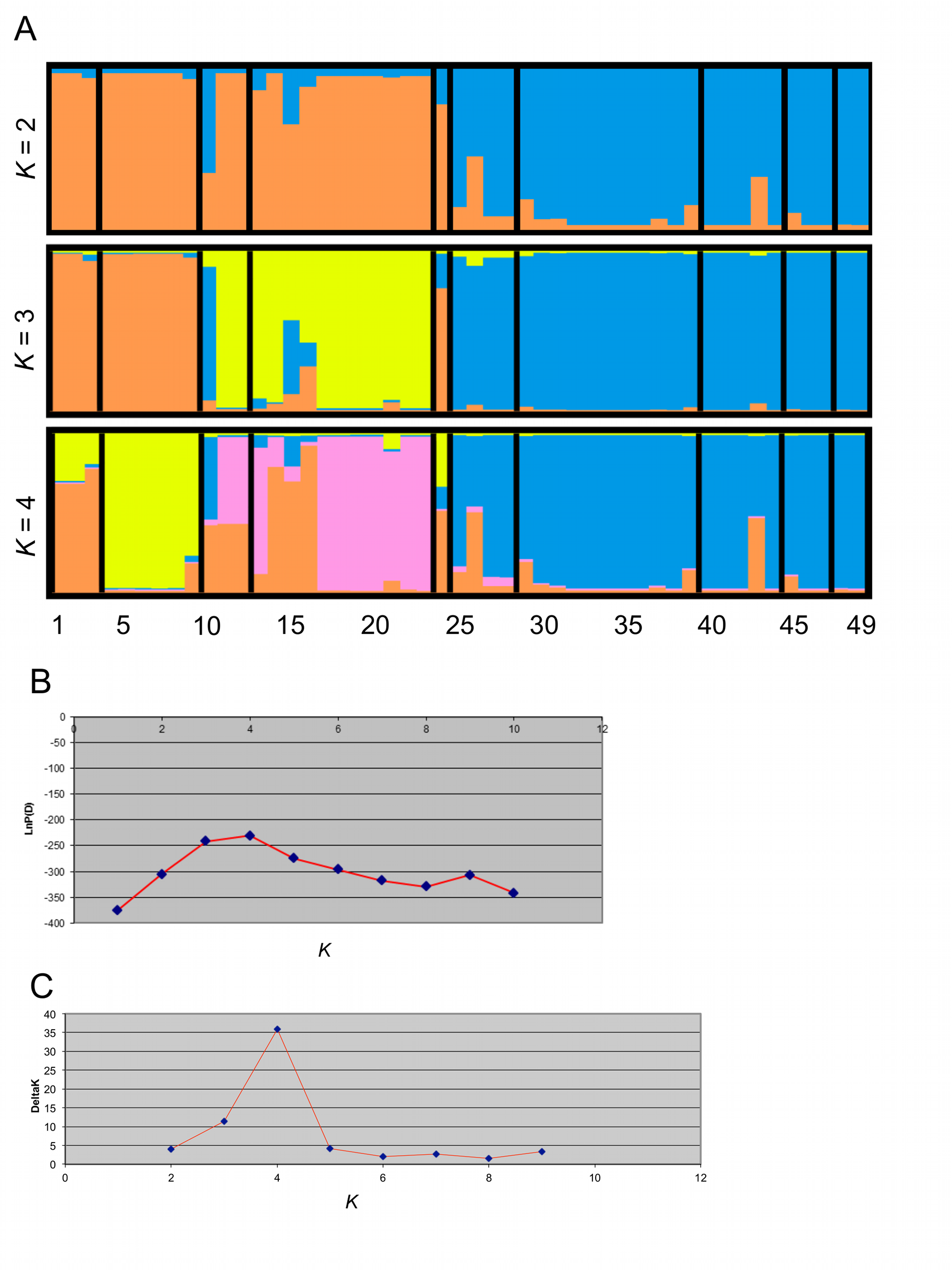

Supplement: Figure S5 — Results of population clustering based on the data of cpDNA and nuclear WER and CHS genes using STRUCTURE software. See the caption for Figure S1 for details. (A) Inference of population structure for the clustering of K = 2, 3, and 4. (B) Mean posterior probability of the data ln P(X|K) over 10 runs for each K-value. (C) Plot of ΔK for each K. (TIF) [file pgen.1002838.s005.tif]
