# Supplementary material for: Recent Loss of Self-Incompatibility by Degradation of the Male Component in Allotetraploid Arabidopsis kamchatica
Source: PLoS Genet. 2012 Jul 26;8(7):e1002838. doi: 10.1371/journal.pgen.1002838 (PMC3405996; doi:10.1371/journal.pgen.1002838)
Supplement: Table S4 — Pattern of intrapopulation segregation in a Kamchatka population from Petropavlovsk Kamchatskii, Mishenaya gora. (DOC) [file pgen.1002838.s009.doc]

Table S4. Pattern of the intra-population segregation in a Kamchatka population, Petropavlovsk Kamchatskii, Mishenaya gora.

| Individuals | *AkSRK-A* | *AkSRK-B* | *AkSRK-C* | *AkSRK-D* | *AkSRK-E* |
| --- | --- | --- | --- | --- | --- |
| 1† | no | yes | yes | yes | no |
| 2 | no | no | yes | yes | no |
| 3 | no | no | yes | yes | no |
| 4 | no | no | yes | yes | no |
| 5 | no | yes | no | yes | no |
| 6 | no | yes | no | yes | no |
| 7 | no | yes | no | yes | no |
| 8 | no | no | yes | yes | no |
| 9 | no | yes | no | yes | no |
| 10 | no | yes | no | yes | no |

Note: Results of PCR-based genotyping are shown. "yes" indicates amplification and "no" indicates no amplification.

† The same individual as No. 33 listed in Table S1.
