# Supplementary material for: Recent Loss of Self-Incompatibility by Degradation of the Male Component in Allotetraploid Arabidopsis kamchatica
Source: PLoS Genet. 2012 Jul 26;8(7):e1002838. doi: 10.1371/journal.pgen.1002838 (PMC3405996; doi:10.1371/journal.pgen.1002838)
Supplement: Table S7 — Intraspecific crosses between two accessions to exclude the possibility that the male components of haplogroup D of the Murodo accession and of haplogroup A of the Biwako accession remain functional. (DOC) [file pgen.1002838.s012.doc]

Table S7. Intraspecific crosses between two accessions to exclude the possibility that the male components of haplogroup D of the Murodo accession and of haplogroup A of the Biwako accession remain functional.

|  | Pollen donor: *A. kamchatica* (4n) | |
| --- | --- | --- |
| Pistil donor: *A. kamchatica* (4n) | Murodo | Biwako |
| Murodo |  | 3/3 |
| Biwako | 4/4 |  |

Note: Numerators denote crosses where more than 20 pollen tubes penetrate the stigma, *i.e.,* compatible reactions. Denominators denote the total number of crosses conducted in each combination.
