# Supplementary material for: Recent Loss of Self-Incompatibility by Degradation of the Male Component in Allotetraploid Arabidopsis kamchatica
Source: PLoS Genet. 2012 Jul 26;8(7):e1002838. doi: 10.1371/journal.pgen.1002838 (PMC3405996; doi:10.1371/journal.pgen.1002838)
Supplement: Text S1 — Population Structure Based on the Software STRUCTURE. (DOC) [file pgen.1002838.s016.doc]

# Supporting Text 1: Population Structure Based on the Software STRUCTURE

In addition to the software InStruct, we used the software STRUCTURE 2.2.3, in which inbreeding is not incorporated [119]. The data were treated as haploid, as recommended for completely selfing species [115] and as commonly performed for predominantly selfing species (e.g., [82,83]). While we did not use chloroplast DNA data for the InStruct analysis, we used them for the STRUCTURE analysis, in addition to homeologous copies of nuclear *CHS* and *WER* genes. The pattern of clustering was overall consistent with that of InStruct, although *K* = 4 was best supported, instead of *K* = 2 (Figure S5). Different estimates for *K* would mean hierarchical population structures [118]. We note that *K* = 3 was best supported instead of *K* = 4 in a previous study [48], with a slightly different sampling of accessions and with an older version of STRUCTURE (version 2.2.3 in this study and version 2.2 in Shimizu-Inatsugi et al. [48]).

Based on the clustering of STRUCTURE, we also investigated the associations between *S*-haplogroups and population structures. See Table S9 for cluster assignments based on *K* = 2, 3 or 4. The distributions of *A. halleri*-derived *S*-haplogroups—*AkSRK-A*, *AkSRK-B* and *AkSRK-C*—are significantly correlated with the population structure (*K* = 4) based on STRUCTURE (Cramer’s coefficient: 0.552; *p* = 9.46 × 10^–10^). Also significant were the correlations with the clustering of *K* = 3 (Cramer’s coefficient: 0.676; *p* = 2.186 × 10^–11^) and even of *K* = 2 (Cramer’s coefficient: 1; *p* = 7.617 × 10^–12^). In contrast, no significant correlation was observed between the *A. lyrata*-derived *S-*haplogroups (*AkSRK-D* and *AkSRK-E*) and population structure (Cramer’s coefficient: 0.172; *p* = 0.442). Furthermore, the correlations were not significant with the clustering of *K* = 3 (Cramer’s coefficient: 0.196; *p* = 0.307) or even of *K* = 2 (Cramer’s coefficient: 0.302; *p* = 0.1093).
